# Biosafety and Fermentation Performance of *Wickerhamomyces anomalus* Starter Cultures in Fully Washed and Modified Whole-Cherry Fermentation of *Coffea canephora*

**DOI:** 10.3390/foods15203721

**Published:** 2026-10-19

**Authors:** Phansiya Thidnithinon, Titiporn Panyachanakul, Siritron Samosorn, Kulvadee Dolsophon, Rossaporn Jiamjariyatam, Wanlapa Lorliam, Ramida Watanapokasin, Nuttika Suwannasai, Sukhumaporn Krajangsang

**Affiliations:** 1 Department of Microbiology, Faculty of Science, Srinakharinwirot University, 114 Sukhumvit 23, Wattana, Bangkok 10110, Thailand; phansiya.ttt@g.swu.ac.th (P.T.);; 2 Department of Science and Bioinnovation, Faculty of Liberal arts and Science, Kasetsart University Kamphaeng Saen Campus, Nakhon Pathom 73140, Thailand; 3 Department of Chemistry, Faculty of Science, Srinakharinwirot University, 114 Sukhumvit 23, Wattana, Bangkok 10110, Thailand; 4 Department of Biochemistry, Faculty of Medicine, Srinakharinwirot University, 114 Sukhumvit 23, Wattana, Bangkok 10110, Thailand

**Keywords:** coffee fermentation, *Wickerhamomyces anomalus*, biosafety evaluation, microbial population, robusta coffee, sensory quality

## Abstract

Fermentation strongly influences coffee quality, yet spontaneous fermentation often results in inconsistent sensory profiles due to uncontrolled microbial dynamics. This study evaluated the biosafety and fermentation performance of two *Wickerhamomyces anomalus* starter cultures, YWP1-3 and UV22-2, for controlled Robusta coffee fermentation. Biosafety was assessed based on antifungal susceptibility, hemolytic activity, and cytotoxicity toward Caco-2 cells. The starters were subsequently applied at different inoculum levels (OD_600_ 15–45) for 24–72 h using fully washed and modified whole-cherry processes. Both strains exhibited γ-hemolysis, but Caco-2 cell viability remained relatively high at low-to-moderate concentrations (≤12.5% *v*/*v*). Controlled inoculation influenced microbial population and physicochemical changes during fermentation; the modified whole-cherry process generally produced higher cup scores and more balanced sensory attributes than the fully washed process. Notably, at the highest inoculum level, *W. anomalus* YWP1-3 enhanced desirable chocolate, raisin, and sweet notes and improved mouthfeel. Overall, the results demonstrate that selected *W. anomalus* starters are associated with changes in culturable microbial populations and fermentation characteristics. Integrating controlled yeast inoculation with modified whole-cherry fermentation represents a promising strategy for improving fermentation consistency and the sensory quality of Robusta coffee.

## 1. Introduction

Coffee is an important agricultural commodity and a major component of the global food and beverage value chain [1]. Among commercially cultivated coffee species, *Coffea canephora* (Robusta coffee) has received increasing attention due to its high productivity, resistance to pests and diseases, and greater tolerance to elevated temperatures and climate variability compared with *Coffea arabica* [2]. In the face of challenges associated with climate change increasing and Arabica suitability in traditional production areas declining, Robusta has been recognized as a strategic crop for maintaining coffee supply stability and supporting the expanding specialty coffee sector. However, despite its agronomic advantages, Robusta coffee is generally associated with lower sensory complexity due to its pronounced bitterness, astringency, earthy characteristics, and higher caffeine and phenolic contents, which often result in lower market value compared with Arabica [3,4].

Recently, with improvements in post-harvest management, fermentation technology, and quality control systems, the perception of Robusta coffee has shifted from a commodity-grade product toward high-quality Fine Robusta [2,5]. In Thailand, Robusta coffee has traditionally been cultivated in the southern regions; however, production areas have now expanded into new regions such as Loei Province, where differences in altitude, climate, and environmental conditions provide opportunities for developing unique sensory profiles. Therefore, optimizing post-harvest processing strategies is essential to enhancing Robusta quality and increasing its competitiveness in specialty coffee markets.

Coffee fermentation is a key post-harvest process that strongly influences final cup quality by modifying the chemical composition of coffee beans through microbial metabolism. During fermentation, microorganisms utilize sugars, pectin, and other mucilage components, leading to the production of organic acids, alcohols, esters, and aroma precursors associated with flavor development [6,7]. Conventional processes, including dry, fully washed, and whole-cherry processing, create distinct fermentation environments due to differences in oxygen availability, water activity, and substrate accessibility. Honey- and whole-cherry-based fermentation systems have recently attracted attention because prolonged contact between coffee beans and mucilage can promote sweetness, body, and flavor complexity. However, spontaneous fermentation remains difficult to control because indigenous microbial communities vary depending on geographical origin, processing conditions, and environmental factors, which frequently result in inconsistent quality and undesirable metabolites [8,9].

To overcome these limitations, controlled fermentation using defined microbial starter cultures has emerged as a promising approach to improving fermentation reproducibility and directing desirable biochemical transformations. Recent studies have demonstrated that selected yeasts can regulate microbial succession, enhance substrate utilization, and influence the formation of flavor-related metabolites during coffee fermentation [10,11,12]. Yeast species including *Saccharomyces cerevisiae*, *Pichia kluyveri*, and *Wickerhamomyces anomalus* have been reported to produce extracellular enzymes and metabolites such as organic acids, higher alcohols, and esters that contribute to fruity, floral, and sweet sensory characteristics.

Recently, controlled semi-anaerobic and whole-cherry fermentation approaches have attracted increasing interest as alternative strategies to enhance microbial control while maintaining complex interactions between coffee cherries and fermenting microorganisms. Unlike conventional washed fermentation, modified whole-cherry or whole-cherry fermentation maintains extended contact among the pulp, mucilage, and beans, providing additional substrates for microbial metabolism. Nevertheless, limited information is available regarding the application of defined yeast starters in Robusta coffee under these fermentation systems, particularly regarding their effects on microbial succession, fermentation dynamics, and sensory quality.

Our previous study identified *Wickerhamomyces anomalus* YWP1-3 as a promising coffee-derived yeast starter capable of improving Arabica coffee fermentation quality. The UV-mutant strain *W. anomalus* UV22-2 was subsequently developed, demonstrating enhanced pectinase and amylase activities, improved substrate utilization ability, and increased production of aroma-related compounds such as furfuryl alcohol and furfuryl acetate [12]. These characteristics suggest the potential application of these strains as functional starter cultures for controlled coffee fermentation; however, their effectiveness in Robusta coffee processing remains undetermined.

Beyond fermentation performance, the safety evaluation of microbial starter cultures has become increasingly important for industrial food applications. Since starter cultures are intentionally introduced into food production systems, they must be evaluated to confirm the absence of potential safety concerns, including antimicrobial resistance, hemolytic activity, toxic metabolite production, and adverse effects on intestinal cells [13,14]. Recent regulatory perspectives emphasize that strain-level safety assessment is required, particularly for microorganisms not previously listed as established food-grade cultures. Therefore, integrating functional performance with biosafety evaluation is necessary for developing microbial starters suitable for commercial coffee fermentation.

We hypothesized that selected safe yeast starter cultures could influence culturable microbial population dynamics during Robusta coffee fermentation and that their effects on fermentation dynamics and quality attributes would depend on the processing system due to differences in substrate availability between fully washed and modified whole-cherry fermentation. Accordingly, this study was conducted to evaluate the biosafety characteristics of *W. anomalus* YWP1-3 and UV22-2 and investigate their effects on microbial dynamics, physicochemical changes, and sensory quality during controlled Robusta coffee fermentation using fully washed and modified whole-cherry processes.

## 2. Materials and Methods

### 2.1. Microbial Starter Cultures Used in Coffee Fermentation

The microbial starter cultures employed in this study consisted of two yeast strains, *Wickerhamomyces anomalus* YWP1-3 and *Wickerhamomyces anomalus* UV22-2 [12]. These strains were originally isolated from coffee processing environments in Doi Pha Tang, Wiang Kaen District, Chiang Rai Province, Thailand, an area characterized by fertile conditions suitable for Arabica coffee cultivation and post-harvest processing. The yeast cultures were maintained as glycerol stocks and stored at −20 °C or −80 °C.

### 2.2. Biosafety Assessment

#### 2.2.1. Preparation of Starter Cultures for Biosafety Testing

*Wickerhamomyces anomalus* strains YWP1-3 and UV22-2 were re-activated using the streak plate technique on Yeast Malt Agar (YMA) (yeast extract, 3 g; malt extract, 3 g; peptone, 5 g; glucose, 10 g; agar, 15 g; distilled water, 1000 mL) (HiMedia Laboratories Pvt. Ltd., Mumbai, India) and incubated at room temperature for 48 h. Subsequently, single colonies were inoculated into Yeast Malt Broth (YMB) (yeast extract, 3 g; malt extract, 3 g; peptone, 5 g; glucose, 10 g; distilled water, 1000 mL) (HiMedia Laboratories Pvt. Ltd., Mumbai, India) and cultivated for 48 h using a rotary shaker without temperature control. After incubation, the cultures were harvested by centrifugation ((TOMY Digital Biology Co., Ltd., Tokyo, Japan) at 25,000 *g* at 10 °C for 10 min to obtain cell pellets. The cells were washed twice with 0.85% (*w*/*v*) sodium chloride solution. Cell density was then adjusted by measuring optical density at 600 nm (OD600) by spectrophotometer (UV-1800, Shimadzu Corporation, Kyoto, Japan) to a value of 30, corresponding to approximately 10^10^–10^11^ viable cells, prior to use in biosafety assays.

#### 2.2.2. Hemolysis Assay

The hemolytic activity of the yeast starter cultures was determined using blood agar. *Wickerhamomyces anomalus* strains YWP1-3 and UV22-2 were first streaked on Yeast Malt Agar (YMA) and incubated at room temperature for 24–48 h. A single colony of each strain was then streaked onto blood agar plates. The inoculated plates were incubated at 25 °C for 24–48 h, after which the hemolytic reaction was examined by observing the appearance of a clear or discolored zone surrounding the colonies. The results were interpreted as follows: β-hemolysis indicated complete hemolysis with a clear zone around the colony, α-hemolysis indicated partial hemolysis with a greenish or brownish zone, and γ-hemolysis indicated no hemolysis and no visible change in the medium surrounding the colony.

#### 2.2.3. Antifungal Susceptibility Test

The antifungal susceptibility of the yeast starter cultures *Wickerhamomyces anomalus* YWP1-3 and UV22-2 was evaluated using a resazurin-based broth microdilution assay in sterile 96-well microplates. The procedure was adapted from the principles described in the Clinical and Laboratory Standards Institute guideline CLSI M27 and the EUCAST reference method E.Def 7.4 for broth microdilution susceptibility testing of yeasts, with modifications to the culture medium, inoculum density, incubation conditions, and resazurin-based endpoint determination [15,16,17]. Itraconazole and clotrimazole stock solutions were prepared in dimethyl sulfoxide (DMSO) at an initial concentration of 20.48 mg/mL. Two-fold serial dilutions of each antifungal agent were prepared across the microplate to obtain final test concentrations ranging from 10.24 to 0.32 mg/ml. After preparation of the dilution series, 50 µL of the final mixture was retained in each well. Subsequently, 50 µL of yeast suspension, adjusted to an OD_600_ of 30 (approximately 10^10^–10^11^ cells/mL, as determined using the established OD_600_–cell concentration relationship); 50 µL of Yeast Malt Broth (YMB); and 50 µL of resazurin solution were added to each test well for a final volume of 200 µL per well. Three control conditions were included. The growth control contained the yeast suspension, YMB, resazurin solution, and an equivalent volume of sterile diluent but no antifungal agent; this control confirmed the ability of the yeast to grow under the assay conditions. The solvent control contained the yeast suspension, YMB, resazurin solution, and DMSO at the same final concentration as in the antifungal-treatment wells but without an antifungal agent; this control was used to verify that the solvent did not inhibit yeast growth. The sterility control contained YMB, resazurin solution, and the corresponding diluent or DMSO but no yeast cells; this control was used to confirm the absence of microbial contamination and non-biological resazurin reduction. The assay was performed using three independent biological replicates for each yeast strain. Each biological replicate was prepared from an independently grown yeast culture and tested in three technical replicate wells for every antifungal concentration and control condition, resulting in nine measurements per condition. The microplates were incubated at 25 °C for 24 h. Yeast metabolic activity was assessed from the color change in resazurin: a change from purple to pink indicated metabolic activity and yeast growth, whereas retention of the purple color indicated growth inhibition. The minimum inhibitory concentration (MIC) was defined as the lowest tested concentration at which no resazurin color change was observed in all technical replicate wells relative to the corresponding growth and solvent controls. MIC values from the three independent biological replicates were reported individually or as the modal MIC and observed range.

#### 2.2.4. Cytotoxicity Assay (MTT Assay)

*Wickerhamomyces anomalus* YWP1-3 and UV22-2 suspensions were adjusted to an optical density of OD_600_ = 30 and separately inoculated into 50 mL of filtered coffee mucilage broth. Uninoculated mucilage broth subjected to the same incubation conditions served as the fermentation-matrix control. The inoculated and uninoculated broths were incubated in a temperature-controlled shaking incubator at 25 °C and 150 rpm. Samples were collected at 0, 24, 48, and 72 h. Three independent fermentation replicates were prepared for each strain and the uninoculated control at each sampling time. At each sampling time, a 50 mL fermentation sample was centrifuged at 25,000× *g* and 10 °C for 10 min. The supernatant was collected, passed through a 0.22 µm membrane filter, stored at 4 °C, and analyzed within one week. Before cell treatment, each filtered supernatant was diluted 1:10 in cell-culture medium. Caco-2 cells (Caco-2; ATCC HTB-37) were cultured at 37 °C in a humidified atmosphere containing 5% CO_2_. The culture medium was then removed, and the cells were exposed to 50 µL of diluted fermentation supernatant for 24 h. Cells maintained in complete culture medium served as the untreated control and were assigned 100% viability. Cells exposed to uninoculated mucilage broth collected at the corresponding incubation times served as matrix controls. Cell-free wells containing the corresponding treatment solutions and MTT reagent (3-(4,5-dimethylthiazol-2-yl)-2,5-diphenyltetrazolium bromide (MTT; Sigma-Aldrich, St. Louis, MO, USA) were included to assess background absorbance and possible interference from the mucilage matrix. After the 24 h of exposure, 10 µL of MTT reagent was added to each well, and the plates were incubated for 4 h at 37 °C. Absorbance was measured at 570 nm as the reference wavelength. Cell viability was calculated as a percentage of the untreated control after subtraction of the appropriate cell-free background absorbance.

### 2.3. Robusta Coffee Fermentation

#### 2.3.1. Yeast Inoculum Preparation

Two strains of *Wickerhamomyces anomalus*, namely YWP1-3 and UV22-2, were used as starter cultures for the fermentation experiments. The strains were initially streaked onto Yeast Malt Agar (YMA) plates and incubated at 25 °C for 24–48 h. The colonies were then transferred into Yeast Malt Broth (YMB) for liquid cultivation. The cultures were incubated in a shaking incubator at 150 rpm at room temperature for 24–48 h. After incubation, a simple streak plate technique was performed again to confirm culture purity. The yeast cells were then harvested by scraping and suspended in 50 mL of sterile 0.85% (*w*/*v*) NaCl solution. The yeast inoculum was prepared by concentrating the cell suspension, followed by serial dilution to obtain measurable OD_600_ values within the range of 0–0.9. The actual OD_600_ was calculated by multiplying the measured absorbance by the dilution factor, and the cell suspension was subsequently adjusted to final OD_600_ values of 15, 30, and 45 before being applied as starter inocula for coffee fermentation. The inoculum was adjusted to OD600 values of 15, 30, and 45, corresponding to estimated viable cell concentrations of approximately 5 × 10^9^–5 × 10^10^, 1 × 10^10^–1 × 10^11^, and 1.5 × 10^10^–1.5 × 10^11^ CFU/mL, respectively. The inoculum was applied at a volume of 50 mL per 8.5 kg of fresh coffee cherries.

#### 2.3.2. Coffee Cherry Preparation

Ripe *Coffea canephora* (Robusta) cherries were harvested from coffee plantations in Loei, Thailand, and transported under refrigerated conditions to Srinakharinwirot University in Bangkok to maintain their freshness. Fully ripe, uniformly red cherries were selected by visual inspection, and underripe (green), overripe (dark-colored or shriveled), insect-damaged, and mechanically damaged cherries were excluded. A flotation test was subsequently performed, and floating cherries were discarded to improve the uniformity of fruit density and quality. Before fermentation, the selected cherries were thoroughly washed with clean water to remove adhering soil, debris, and surface contaminants.

#### 2.3.3. Fully Washed Process

Coffee cherries were washed with clean water 2–3 times to remove dirt and impurities, and floating cherries were discarded. The cherries were then mechanically depulped to remove the coffee pulp. The depulped beans were washed repeatedly until the washing water was clear. At this stage, the mucilage layer remained attached to the parchment coffee beans, indicating they were ready to undergo fermentation under the designed experimental conditions. Fermentation was conducted in 25 L tanks containing 10 L of water; the tanks were sealed with lids throughout the fermentation period. Three independent fermentation units were prepared for each treatment using coffee cherries from a single harvest batch. Consequently, variability among harvest batches and processing dates was not evaluated. All fermentation units were maintained separately under identical conditions at 20 °C.

#### 2.3.4. Modified Whole-Cherry Process

For the whole-cherry process, coffee cherries were first washed with clean water 2–3 times to remove impurities, and floating cherries were discarded. The cleaned coffee cherries were then placed into sterile plastic bags at a weight of 8.5 kg per bag. Unlike the fully washed process, the cherries were fermented without prior depulping. Excess air was expelled from each bag, after which it was sealed tightly. At 24 h intervals, each bag was shaken to mix the cherries thoroughly before being opened. The bags were then kept open for 10 min and subsequently resealed after the excess air had been expelled. Three independent fermentation units were prepared for each treatment using cherries from the same harvest batch; therefore, the experiment did not account for variation among harvest batches or processing dates. Fermentation was carried out at 20 °C, with each unit maintained separately under identical conditions. At each designated sampling time, samples were collected separately from the individual fermentation units for subsequent analyses. After completion of the predetermined fermentation period, the cherries were depulped, and the beans were washed until the washing water was clear.

#### 2.3.5. Experimental Design

The prepared yeast inocula were added to the Robusta coffee samples prepared as described above. Fermentation was conducted in fermentation tanks (fully washed process) and sterile plastic bags (whole-cherry process) at 20 °C. For each fermentation process, two yeast strains were tested at 3 inoculum concentrations (OD_600_ = 15, 30, and 45). A control treatment without yeast inoculation was also included for each fermentation process (Table 1). Three independently prepared control fermentation units were included for each process and handled in parallel with the OD15, OD30 and OD45 treatment sets. The control data were not duplicated or reused across treatment sets. The experimental fermentation process lasted for 72 h in total.

In the modified whole-cherry fermentation process, the cherries were fermented under oxygen-limited conditions with periodic opening and manual mixing at 20 °C at 24, 48, and 72 h (Table 1). During whole-cherry fermentation, the fermentation bags were opened at 24 h intervals, and the materials were manually stirred to maintain homogeneity throughout the fermentation period. After fermentation, the cherries were depulped to remove the outer skin and pulp. The remaining mucilage was subsequently removed by washing with clean water, followed by drying until the coffee beans reached the desired moisture content (approximately 9–11%). The dried parchment coffee was then stored under appropriate conditions for further analysis.

### 2.4. Sampling of Robusta Coffee for Microbiological Analysis

In this study, Robusta coffee samples were collected according to the fermentation conditions described in Table 1. Samples were collected at four fermentation time points: 0, 24, 48, and 72 h. The experimental design included two fermentation processes (fully washed and whole-cherry), three inoculum concentrations (OD_600_ = 15, 30, and 45), and two yeast strains, including a control treatment without inoculum. For each treatment, three 20 g subsamples were collected from each fermentation vessel (from the tank for the fully washed process or bags for the modified whole-cherry process). Each subsample consisted of parchment coffee for the fully washed process or whole coffee cherries for the modified whole-cherry process and was divided into two portions for the measurement of soluble solids (°Brix) and pH and the enumeration of culturable microbial populations.

#### 2.4.1. Measurement of Soluble Solids Content and pH

For the fully washed fermented samples, the fermentation liquid was collected directly from each treatment and used for the determination of the soluble solids content and pH. For the whole-cherry fermentation samples, the fermentation liquid was obtained by manually squeezing the coffee cherries to extract the mucilage-associated liquid prior to analysis. All fermentation liquid samples were directly analyzed without dilution. The soluble solids content was measured using a digital refractometer and expressed as °Brix, while the pH was determined using a pH meter (Mettler-Toledo GmbH, Greifensee, Switzerland). All measurements were performed following 0, 24, 48, and 72 h of fermentation, and the obtained values were recorded.

#### 2.4.2. Determination of Microbial Populations During Robusta Coffee Fermentation

For microbial population analysis, a 10 g sample of coffee parchment (fully washed) or coffee cherries (whole-cherry process) from each treatment was squeezed to obtain fermentation liquid and mixed with 90 mL of sodium chloride solution. Serial dilutions ranging from 10^−1^ to 10^−7^ were prepared. Aliquots were spread onto four different culture media using the spread plate method to enumerate various microbial populations. Serially diluted fermentation liquid samples (10^−1^–10^−7^) were used for microbial enumeration. For total bacterial counts, 100 µL of each dilution was spread onto Plate Count Agar (PCA; HiMedia Laboratories Pvt. Ltd., Mumbai, India) plates and incubated at 37 °C for 24 h. Total yeast populations were determined by spreading 100 µL of diluted samples onto Yeast Malt Agar supplemented with chloramphenicol (YMA+; HiMedia Laboratories Pvt. Ltd., Mumbai, India), followed by incubation at 25 °C for 24–48 h. Lactic acid bacteria (LAB) were enumerated by plating 100 µL of the diluted samples onto Lactobacillus MRS agar (MRS; HiMedia Laboratories Pvt. Ltd., Mumbai, India) plates. The plates were incubated under anaerobic conditions in an anaerobic jar at 30 °C for 5 days. For mold enumeration, 100 µL of diluted samples were spread onto Potato Dextrose Agar (PDA; HiMedia Laboratories Pvt. Ltd., Mumbai, India) plates and incubated at 30 °C for 7 days. After incubation, colonies were counted and the results were expressed as log CFU/g.

### 2.5. Post-Fermentation Processing of Robusta Coffee

After fermentation under each condition (24, 48, and 72 h), the coffee beans were washed with clean water to remove the remaining mucilage. The beans were then sun-dried until the moisture content decreased to approximately 9–11%. Once the desired moisture level was achieved, the beans from each treatment were packed in moisture-barrier bags (GrainPro, GrainPro, Inc., Washington, DC, USA) and stored at room temperature in a dry environment until sensory evaluation.

### 2.6. Sensory Evaluation of Roasted Robusta Coffee

Sensory evaluation was carried out to determine the effects of the fermentation treatments on the cup quality of *Coffea canephora*. The evaluation was performed by five trained and certified Q Robusta Graders experienced in the sensory assessment of Fine Robusta coffee. Sample preparation and cupping were conducted in accordance with the Fine Robusta Cupping Protocol developed by the Coffee Quality Institute (CQI). Green coffee beans from each treatment were roasted using a laboratory-scale sample roaster to a medium to medium–dark roast level. The target roast color was approximately Agtron 48 for whole beans and 78 ± 1 for ground coffee. Roasting was completed within 9–14 min, and the beans were immediately air-cooled without water quenching. The roasted samples were stored in airtight containers at room temperature and allowed to rest for 8–24 h before cupping. All samples were assigned randomized three-digit codes and presented to the graders under blinded conditions. For each sample, five cups were prepared to permit the assessment of cup uniformity. Whole roasted beans were weighed at a ratio of 8.75 ± 0.25 g per 150 mL of water and ground no more than 15 min before infusion. The grind size was slightly coarser than that used for paper-filter brewing, with approximately 70–75% of the particles passing through a U.S. Standard No. 20 mesh sieve. Each cup was infused with 150 mL of clean, odor-free water at approximately 93 °C. The coffee grounds were allowed to steep undisturbed for 4 min, after which the crust was broken by stirring three times while the wet aroma was evaluated. These preparation conditions conform to the published Fine Robusta protocol. Sensory evaluation began when the beverage temperature decreased to approximately 70 °C and continued as the samples cooled. The attributes assessed using the Fine Robusta Cupping Form were Fragrance/Aroma, Flavor, Aftertaste, Salt/Acid Aspect Ratio, Bitter/Sweet Aspect Ratio, Mouthfeel, Balance, Uniform Cups, Clean Cups, and Overall. Quality attributes were scored from 6.00 to 10.00 in increments of 0.25, where 6.00, 7.00, 8.00, and 9.00 represented good, very good, fine, and outstanding quality, respectively. Faults were recorded and deducted from the total score to obtain the final cup score. Coffees with final scores of ≥80 points were classified as Fine Robusta. The graders evaluated the coded samples independently and rinsed their palates with drinking water between samples. Three independent cupping sessions were conducted on separate occasions. Within each session, five cups were prepared per treatment. For statistical analysis, the score assigned by each grader to a treatment in each independent session was considered an individual sensory observation, and the results were expressed as the mean ± standard deviation of the grader and session scores.

### 2.7. Statistical Analysis

Statistical analyses were performed using IBM SPSS Statistics software (version 23.0, IBM Corp., Armonk, NY, USA). Significant differences among treatments were evaluated using two-way ANOVA, followed by Fisher’s least significant difference (LSD) post hoc test. A *p* value of less than 0.05 was considered statistically significant. Pearson correlation analysis was performed to evaluate the relationships among physicochemical parameters (pH and °Brix), culturable microbial populations (total bacteria, total yeast, lactic acid bacteria, and total mold), and total cupping scores. The analysis was conducted separately for the Fully Washed and modified whole-cherry processes to account for differences in fermentation conditions between the two processing methods. Pearson correlation coefficients (r) were calculated using treatment-matched observations across fermentation conditions. Statistical significance was determined at *p* < 0.05. Correlation matrices were visualized as heatmaps, with coefficients ranging from −1 to +1, representing negative and positive linear relationships, respectively. Statistical significance was indicated as *p* < 0.05, *p* < 0.01, and *p* < 0.001. Correlation analysis was used to identify associations among variables and was not interpreted as evidence of causal relationships.

## 3. Results

### 3.1. Biosafety Assessment of Yeasts for Coffee Fermentation

#### 3.1.1. Antimicrobial Susceptibility Test and Hemolytic Activity

The antifungal susceptibility testing of the yeast starter cultures *Wickerhamomyces anomalus* YWP1-3 and UV22-2 showed a one-dilution difference in their MIC values for clotrimazole, whereas identical MIC values were observed for itraconazole. For clotrimazole, YWP1-3 exhibited an MIC of 1.28 mg/mL compared with 2.56 mg/mL for UV22-2, indicating that YWP1-3 was inhibited at a twofold lower concentration under the conditions used in this assay. For itraconazole, both strains exhibited an MIC of 5.12 mg/mL, indicating comparable in vitro responses to this antifungal agent. However, species-specific clinical breakpoints and epidemiological cutoff values have not been established by CLSI or EUCAST for itraconazole or clotrimazole against *W. anomalus*. Therefore, these MIC values are presented as descriptive in vitro findings and cannot be used to classify the strains as susceptible, intermediate, or resistant. Although the results provide preliminary information on the antifungal susceptibility profiles of the two starter cultures, further testing using standardized reference methods and additional antifungal agents is required before conclusions regarding antifungal resistance or biosafety can be drawn.

The hemolytic activity of the *Wickerhamomyces anomalus* strains YWP1-3 and UV22-2 was evaluated using blood agar plates. After incubation, neither strain showed visible clear or discolored zones surrounding the colonies. The agar medium remained unchanged, indicating the absence of red blood cell lysis. Based on the hemolysis classification, both YWP1-3 and UV22-2 were identified as γ-hemolytic, which indicates no hemolytic activity. This result suggests that neither strain exhibits the ability to lyse red blood cells under the tested conditions. The absence of hemolytic activity supports the safety of both yeast strains for potential application as starter cultures in coffee fermentation.

#### 3.1.2. Cytotoxicity of *W. anomalus* YWP 1-3 Fermentation Supernatant in Caco-2 Cells

**Figure 1:**
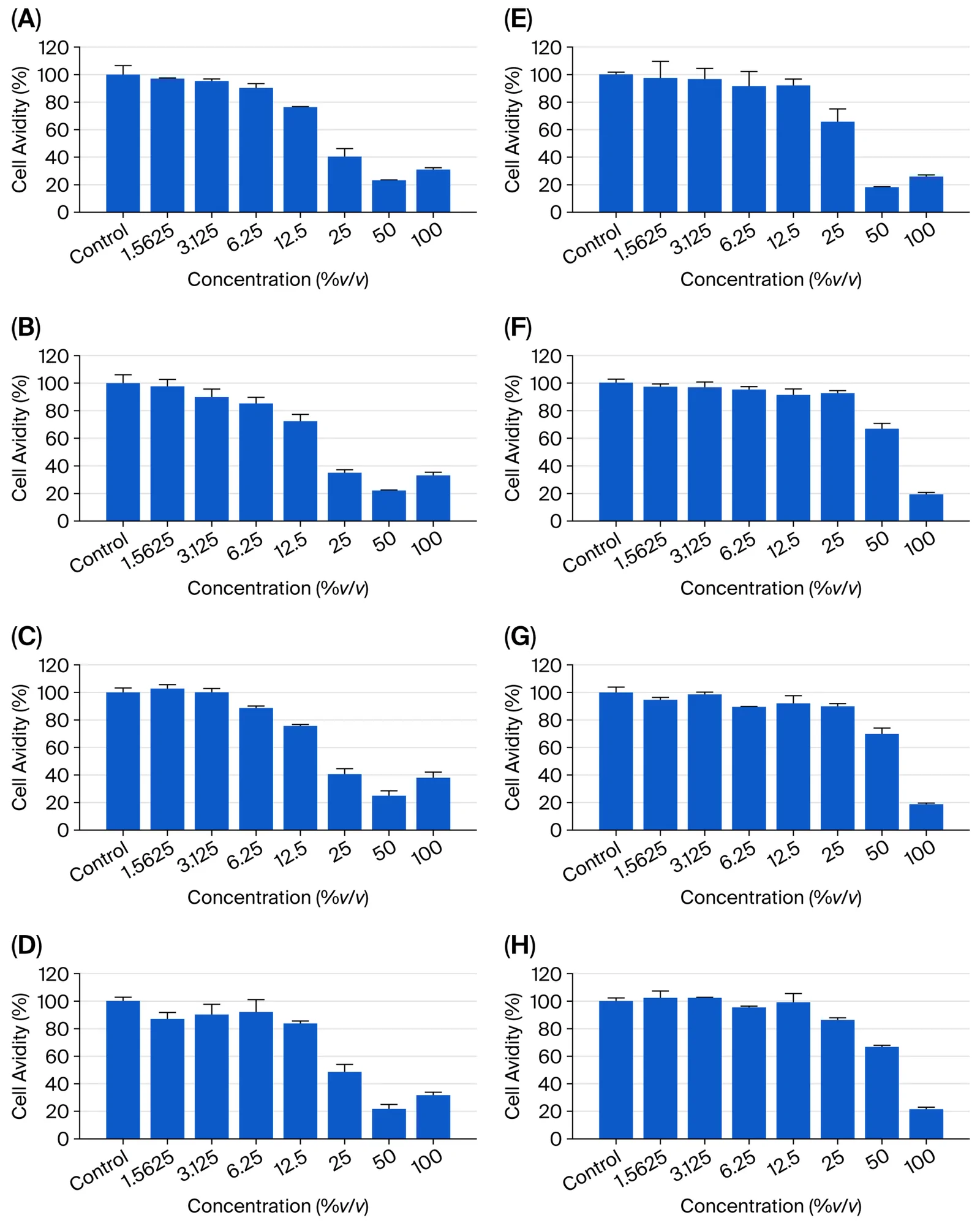
Cytotoxicity assessment of fermentation samples derived from YWP1-3 using Caco-2 cells. Control without starter at 0 h (**A**), 24 h (**B**), 48 h (**C**) and 72 h (**D**). Cell supernatant of strain YWP1-3 at 0 h (**E**), 24 h (**F**), 48 h (**G**), and 72 h (**H**).

The cytotoxicity assessment of fermentation samples derived from YWP1-3 using Caco-2 cells demonstrated a clear concentration-dependent decrease in cell viability across all fermentation time points (Figure 1). A pronounced reduction in cell viability was observed at concentrations of 25% (*v*/*v*) and above when compared with the control group. Although cell viability remained relatively high at low to moderate concentrations (≤12.5% *v*/*v*), ranging from approximately 85 to 100%, a slight decline in viability was already observable at 12.5% (*v*/*v*) at certain time points, particularly when the fermentation duration increased to 48 and 72 h. At a concentration of 25% (*v*/*v*), a moderate reduction in cell viability was detected, whereas at higher concentrations of 50% and 100% (*v*/*v*), cell viability decreased markedly at all time points. These findings indicate that Caco-2 cells are relatively sensitive to the fermentation products generated by YWP1-3 at high concentrations. Although the fermentation time increased, the observed cytotoxic effect was more strongly associated with sample concentration than with fermentation duration. However, the increased cytotoxicity observed when using cell supernatant at higher concentrations showed no significant difference compared with the control experiment without the addition of YWP1-3. Overall, the YWP1-3 fermentation samples exhibited a clear cytotoxic effect at concentrations of 25% (*v*/*v*) and above, with a consistent dose-dependent cellular response pattern.

**Figure 2:**
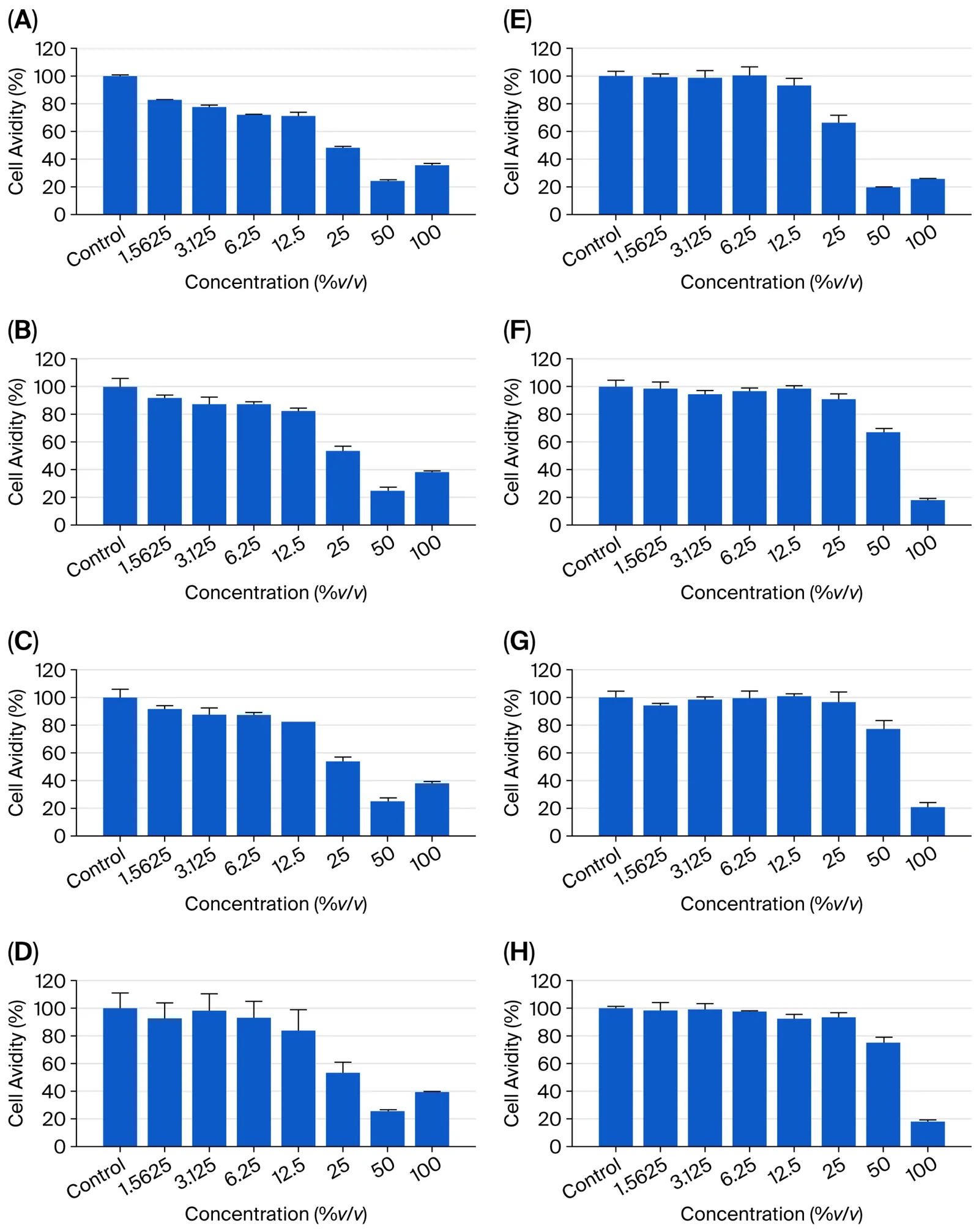
Cytotoxicity assessment of fermentation samples derived from UV22-2 using Caco-2 cells. Control without starter at 0 h (**A**), 24 h (**B**), 48 h (**C**), and 72 h (**D**). Cell supernatant of strain UV22-2 at 0 h (**E**), 24 h (**F**), 48 h (**G**), and 72 h (**H**).

In the case of UV22-2, the cytotoxicity results for Caco-2 cells indicated that cell viability remained high at low to moderate concentrations (≤12.5% *v*/*v*) for all fermentation time points (Figure 2). The majority of viability values ranged between approximately 90 and 100%, showing less variability compared with YWP1-3. Although a reduction in cell viability began to appear at a concentration of 25% (*v*/*v*), the decrease occurred gradually, and at some time points the viability remained relatively high (around 70–80%) before a pronounced decline at higher concentrations of 50% and 100% (*v*/*v*). These results suggest that Caco-2 cells exhibit slightly greater tolerance to fermentation products derived from UV22-2 at moderate concentrations. Furthermore, extending the fermentation time from 0 to 72 h did not result in a notable increase in cytotoxicity within the low-to-moderate concentration range, indicating the relative safety of UV22-2 for intestinal epithelial cells. Overall, qualitative comparison suggests that UV22-2 exhibited slightly lower cytotoxicity toward Caco-2 cells than YWP1-3 at moderate concentrations, and both strains showed pronounced effects on cell viability only at high concentrations.

### 3.2. Evaluation of Yeast Starter Cultures for Improving the Quality of Robusta Coffee

#### 3.2.1. Changes in Soluble Solids (°Brix) and pH During Whole-Cherry Fermentation

**Figure 3:**
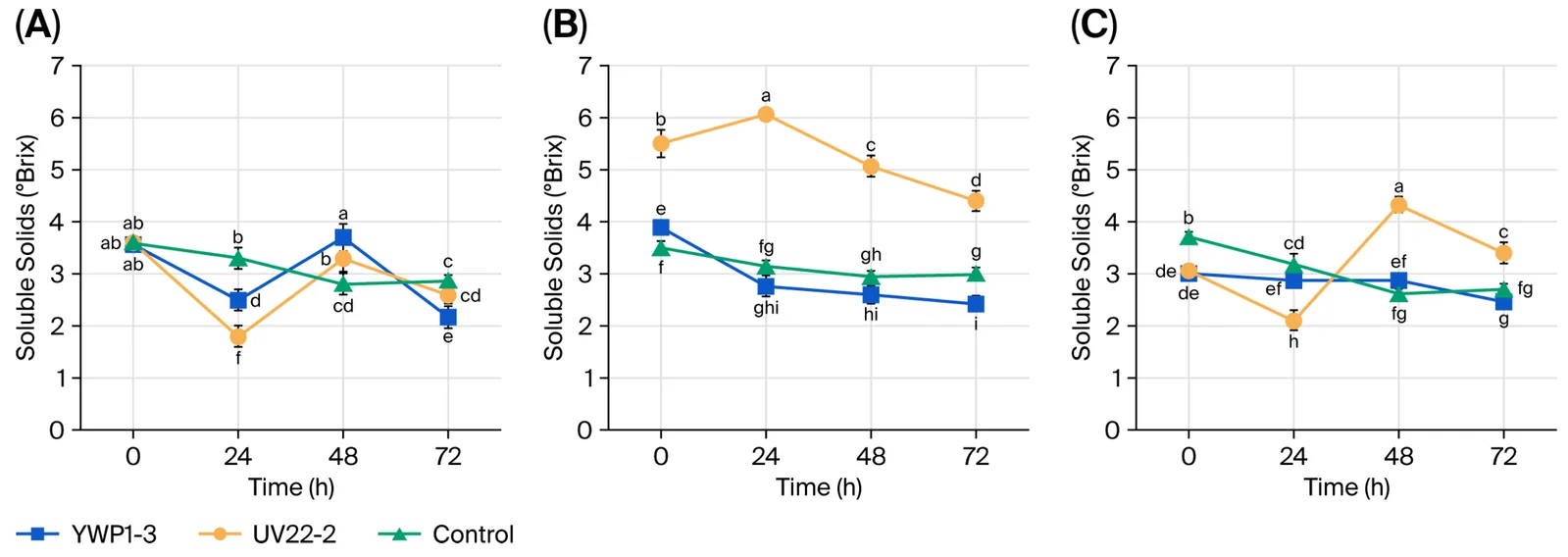
Changes in soluble solids (°Brix) during Robusta coffee fermentation using the whole-cherry process at different yeast inoculum concentrations (OD15 (**A**), OD30 (**B**), and OD45 (**C**)). Data represent mean ± standard deviation (*n* = 9). Different letters indicate statistically significant differences among fermentation times at the same starters (*p* < 0.05).

Changes in soluble solids (°Brix) during Robusta coffee fermentation via the whole-cherry process reflect the utilization of soluble sugars and organic compounds by microorganisms in the fermentation system (Figure 3). Overall, °Brix values tended to decrease during the early stage of fermentation (0–24 h) across all inoculum concentrations, followed by slight fluctuations during the later stages (48–72 h). The extent of this decrease varied depending on the yeast inoculum concentration and strain used. At an inoculum concentration of OD15, the initial °Brix values ranged from approximately 1.6 to 3.7, with the control treatment producing the highest initial value. During the first 24 h of fermentation, the °Brix values decreased in all treatments, with the most pronounced reduction observed for the UV22-2 treatment. After 48 h, the YWP1-3- and UV22-2-treated samples showed a slight increase compared with the values observed at 24 h, whereas the control continued to decrease slightly. At 72 h, the °Brix value in the YWP1-3-treated sample reached the lowest level among all samples, indicating continued utilization of soluble substrates during the final stage of fermentation. At an inoculum concentration of OD30, the initial °Brix values ranged from approximately 2.0 to 3.9, with the YWP1-3-treated sample showing the highest initial value. After 24 h, a decrease in °Brix was observed for all treatments, particularly in the YWP1-3 sample. At 48 h, the UV22-2-treated samples showed a slight increase in °Brix, whereas the YWP1-3 and control samples’ values remained relatively stable or continued to decrease. After 72 h, the UV22-2-treated samples exhibited slightly higher °Brix values than those that underwent other treatments, while the YWP1-3 samples showed the lowest value, suggesting differences in substrate utilization between yeast strains. At an inoculum concentration of OD45, the initial °Brix values ranged from approximately 2.1 to 4.2. A noticeable decrease was observed during the first 24 h, particularly in the UV22-2 group. At 48 h, the UV22-2-treated sample exhibited a marked increase in °Brix compared with the 24 h value, while the YWP1-3 and control samples showed decreasing or relatively stable trends. After 72 h, the °Brix value for the YWP1-3-treated samples decreased to the lowest level, whereas the UV22-2 and control samples exhibited similar values. Overall, the decrease in °Brix during the first 24 h across all inoculum concentrations suggests active microbial utilization of soluble substrates during the early stage of whole-cherry fermentation.

#### 3.2.2. Changes in pH During Robusta Coffee Fermentation via the Whole-Cherry Process

**Figure 4:**
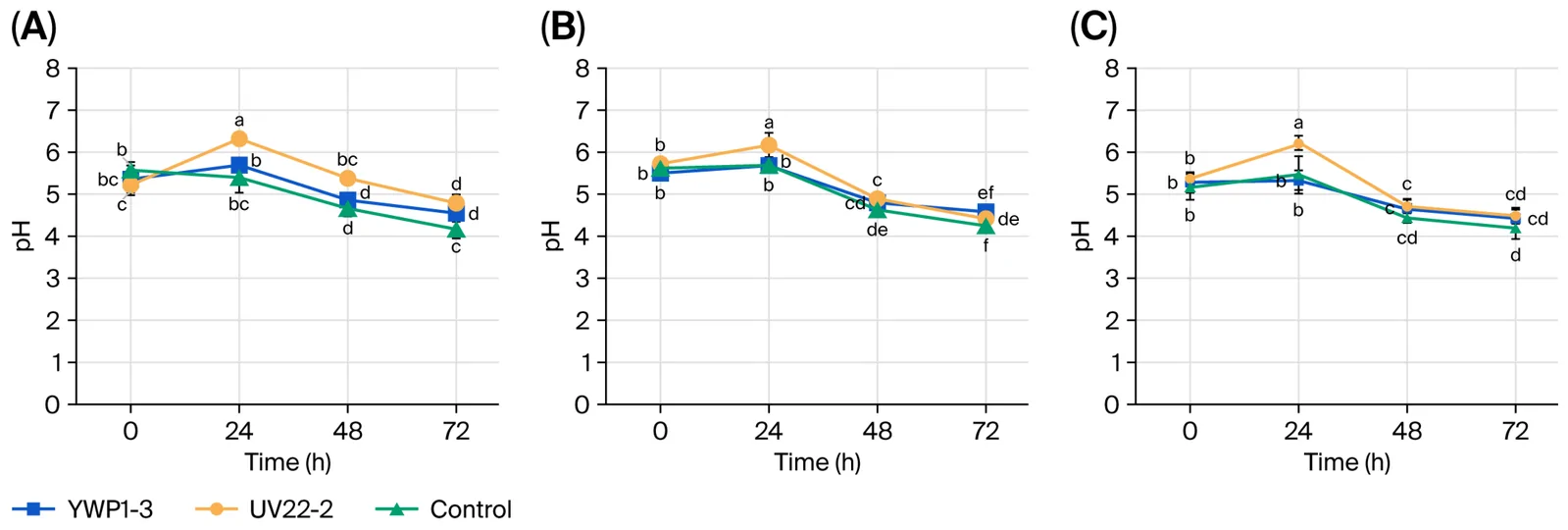
Changes in pH value during Robusta coffee fermentation using the whole-cherry process at different yeast inoculum concentrations (OD15 (**A**), OD30 (**B**), and OD45 (**C**)). Data represent mean ± standard deviation (*n* = 9). Different letters indicate statistically significant differences among fermentation times at the same starters (*p* < 0.05).

Changes in pH during Robusta coffee fermentation showed a general decreasing trend over the fermentation period across all yeast inoculum concentrations (Figure 4). Overall, a slight increase in pH was observed during the first 24 h for some treatments, followed by a marked decrease at 48 and 72 h, indicating the accumulation of organic acids in the fermentation system. At an inoculum concentration of OD15, the initial pH values ranged from approximately 5.2 to 5.6 at 0 h. The YWP1-3 treatment and control sample exhibited similar initial pH values, while the UV22-2 samples showed a slightly lower value. During the first 24 h, the pH following UV22-2 treatment increased markedly to approximately 6.4, whereas the other treatments resulted in only a slight increase. After 48 h, the pH decreased significantly following all treatments, ranging from approximately 4.5 to 5.3. The pH further declined at 72 h, reaching values of approximately 4.1–4.6, representing the lowest pH levels observed during fermentation at this inoculum concentration. At an inoculum concentration of OD30, the initial pH values ranged from approximately 5.4 to 5.7. A slight increase in pH was observed during the first 24 h, particularly following the UV22-2 treatment, which reached approximately 6.0. Subsequently, a pronounced decrease in pH was observed at 48 h across all treatments, with values ranging from approximately 4.5 to 4.9. The pH continued to decline at 72 h, reaching approximately 4.1–4.5, which was comparable to the values observed at the OD15 inoculum level.

#### 3.2.3. Changes in Soluble Solids (°Brix) During Robusta Coffee Fermentation via the Fully Washed Process

**Figure 5:**
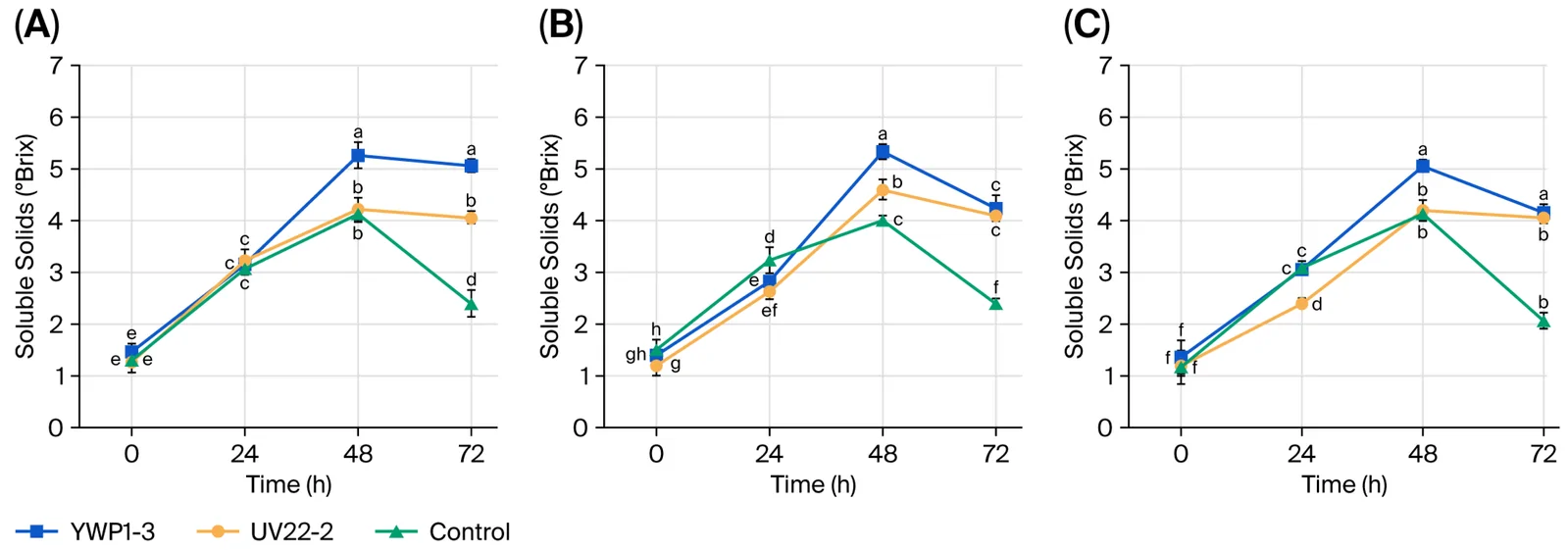
Changes in soluble solids (°Brix) during Robusta coffee fermentation via the fully washed process at different yeast inoculum concentrations (OD15 (**A**), OD30 (**B**), and OD45 (**C**)). Data represent mean ± standard deviation (*n* = 9). Different letters indicate statistically significant differences among fermentation times at the same starters (*p* < 0.05).

The changes in soluble solids (°Brix) during the fully washed fermentation process, as shown in Figure 5, showed a noticeably different pattern from that observed in the whole-cherry process system, particularly during the last stage of fermentation (72 h). Notably, the yeast-inoculation treatments resulted in a pronounced increase in °Brix, whereas the control sample showed only minor fluctuations throughout the fermentation period. At an inoculum concentration of OD15, the initial °Brix values were similarly low across all treatments. During the first 24 and 48 h of fermentation, a slight increase in °Brix was observed following all treatments, suggesting gradual changes in the composition of soluble compounds in the fermentation system. However, a substantial increase was observed at 72 h in the yeast-inoculated samples. The YWP1-3-treated sample exhibited the highest °Brix value (approximately 5.3), followed by UV22-2 (approximately 4.4). In contrast, the control showed a slight decrease and remained at relatively low °Brix levels throughout the fermentation process. A similar trend was observed at an inoculum concentration of OD30. The °Brix values increased gradually during the first 48 h and showed a marked increase at 72 h in the yeast-inoculated samples. The YWP1-3 and UV22-2 samples exhibited comparable °Brix values of approximately 4.0, whereas the control maintained relatively low values with only minor variations. At an inoculum concentration of OD45, a consistent pattern was also observed. The °Brix values increased slightly during the first 24 and 48 h and then increased markedly at 72 h in the yeast-inoculated samples, reaching values close to 4.0. In contrast, the control maintained relatively low and stable °Brix values throughout the fermentation period. Overall, the fully washed fermentation process demonstrated that yeast inoculation significantly increased °Brix during the later stages of fermentation, whereas the controls exhibited minimal changes.

#### 3.2.4. Changes in pH During Robusta Coffee Fermentation via the Fully Washed Process

**Figure 6:**
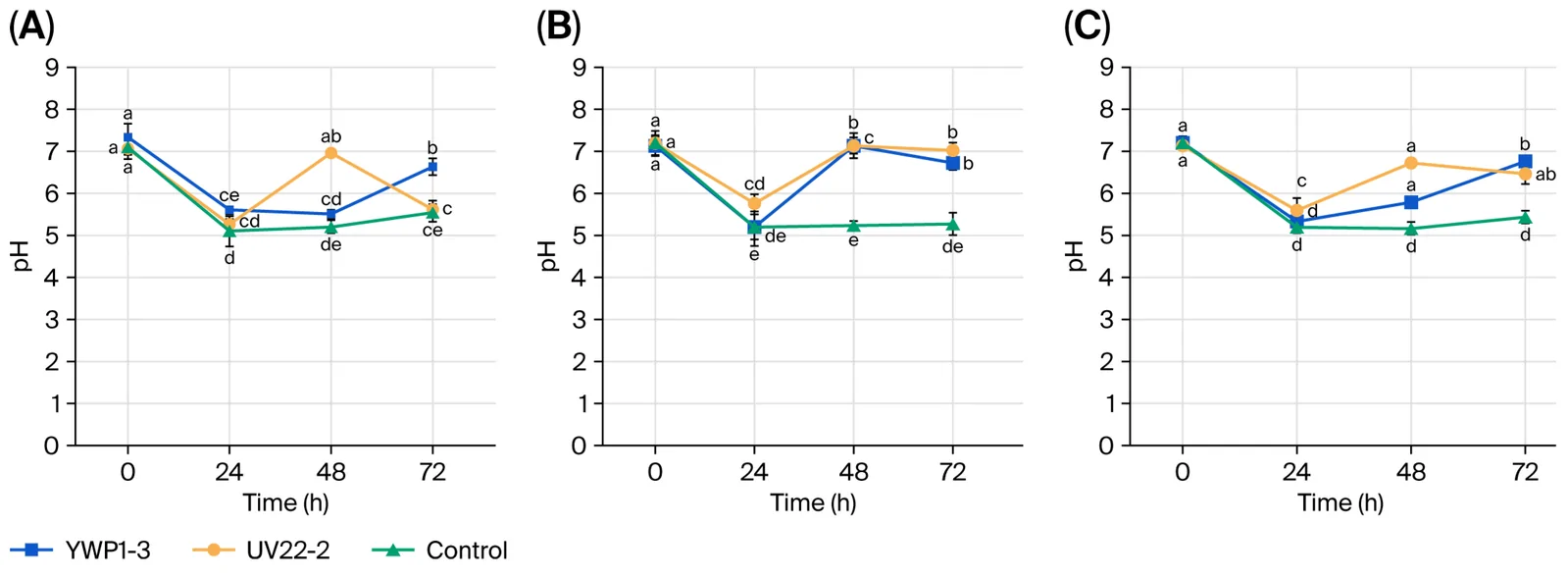
Changes in pH value during Robusta coffee fermentation using the fully washed process at different yeast inoculum concentrations (OD15 (**A**), OD30 (**B**), and OD45 (**C**)). Data represent mean ± standard deviation (*n* = 9). Different letters indicate statistically significant differences among fermentation times at the same starters (*p* < 0.05).

The pH changes observed during the fully washed fermentation process, as shown in Figure 6, showed a pattern noticeable different from that of the whole-cherry process, particularly in the mid-stage of fermentation (48 h) where noticeable fluctuations occurred. In contrast to the whole-cherry fermentation system, the pH during fully washed fermentation did not continuously decrease to very low levels toward the end of fermentation. At an inoculum concentration of OD15, the initial pH values ranged from approximately 7.0 to 7.2 across all treatments. During the first 24 h, the pH decreased markedly in all treatments to approximately 5.0–5.7. However, at 48 h, the UV22-2-treated sample exhibited a substantial increase in pH to approximately 7.0, whereas the YWP1-3 sample remained relatively stable or increased slightly. In contrast, the control sample value remained at a lower level, close to a pH of 5.0. At 72 h, the pH of the YWP1-3-treated sample increased further, while the UV22-2-treated sample showed a decrease, and the control exhibited only a slight increase, indicating a fluctuating fermentation environment under the fully washed condition. At an inoculum concentration of OD30, the initial pH values were similar to those observed at OD15 (approximately 7.0–7.2). A pronounced decrease was observed during the first 24 h, with pH values declining to approximately 5.0–5.7. Subsequently, at 48 h, the yeast-inoculated samples exhibited an increase in pH, particularly those following UV22-2 treatment, whereas the control remained at a lower level (below approximately 5.2). By 72 h, the pH values in the yeast-inoculated samples increased or remained stable at approximately 6.5–6.8, while the pH of the control sample remained distinctly lower. A similar trend was observed at an inoculum concentration of OD45. The pH values decreased during the first 24 h and subsequently increased noticeably at 48 h in the yeast-inoculated samples. At 72 h, the pH values of the yeast-inoculated samples remained clearly higher than those of the control samples, whereas the controls exhibited only a slight increase from the values observed at 24 h. Overall, the fully washed fermented samples showed an initial decrease in pH during the first 24 h, followed by stabilization or partial recovery during the later stages of fermentation. This pattern contrasts with the continuous pH decline typically observed during whole-cherry fermentation. The yeast-inoculated samples tended to exhibit higher or more fluctuating pH values over 48–72 h, whereas the control pH values remained lower and more stable toward the end of fermentation.

### 3.3. Evaluation of Microbial Dynamics During Robusta Coffee Fermentation via the Fully Washed Process

#### 3.3.1. Changes in Total Culturable Bacterial Population During Robusta Coffee Fermentation via the Fully Washed Process

**Figure 7:**
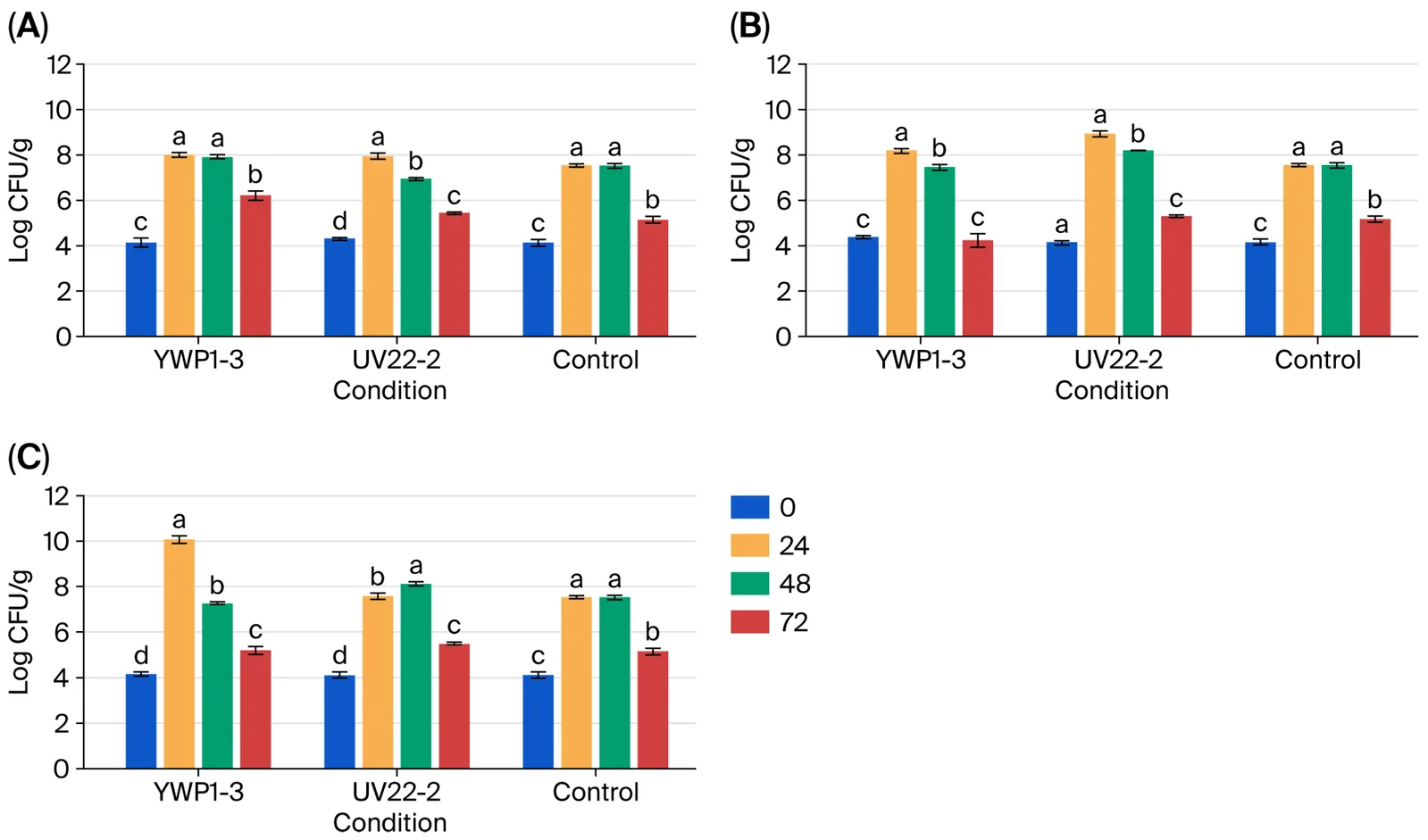
The dynamics of total bacterial populations on PCA during the fully washed fermentation process at different yeast inoculum concentrations (OD15 (**A**), OD30 (**B**), and OD45 (**C**)). Data represent mean ± standard deviation (*n* = 9). Different letters indicate statistically significant differences among fermentation times at the same starters (*p* < 0.05).

The dynamics of total culturable bacterial populations during fully washed fermentation showed a similar pattern across all yeast inoculum concentrations (Figure 7). In general, bacterial populations increased rapidly during the first 24 h of fermentation, followed by a gradual decline over 48–72 h. It should be noted that the detected bacteria were considered indigenous microorganisms, as the inoculated starter cultures consisted of yeast rather than bacterial strains.

At an inoculum concentration of OD15, the initial bacterial population ranged from approximately 4.1 to 4.3 log CFU/g across all treatments. After 24 h of fermentation, bacterial populations increased markedly in all groups, reaching approximately 7.5–8.0 log CFU/g, representing the highest levels observed at this inoculum concentration. At 48 h, the bacterial counts remained relatively high following some treatments but began to show a declining trend. By 72 h, the populations had decreased noticeably to approximately 5.0–6.2 log CFU/g. At an inoculum concentration of OD30, the initial bacterial counts were similar to those observed at OD15 (approximately 4.1–4.3 log CFU/g). After 24 h, the bacterial populations increased substantially, particularly under the UV22-2 treatment, exhibiting the highest counts at this level (approximately 8.8–9.0 log CFU/g). Subsequently, the bacterial counts either remained relatively stable or decreased slightly at 48 h, followed by a more pronounced decline at 72 h, reaching approximately 4.2–5.2 log CFU/g. At an inoculum concentration of OD45, this pattern was even more pronounced. Bacterial populations increased sharply during the first 24 h, with the YWP1-3-treated sample reaching the highest value of approximately 10 log CFU/g, clearly exceeding the levels observed at lower inoculum concentrations. After 48 h, bacterial populations declined under some treatments but remained relatively high. By 72 h, the counts decreased further to approximately 5.0–5.5 log CFU/g, approaching levels similar to those observed under the other treatments. Overall, the fully washed fermentation process demonstrated a rapid proliferation of indigenous bacteria during the first 24 h, corresponding to the active microbial phase of fermentation. This was followed by a decline in bacterial populations over 48–72 h.

#### 3.3.2. Changes in Yeast Population During Robusta Coffee Fermentation via the Fully Washed Process

**Figure 8:**
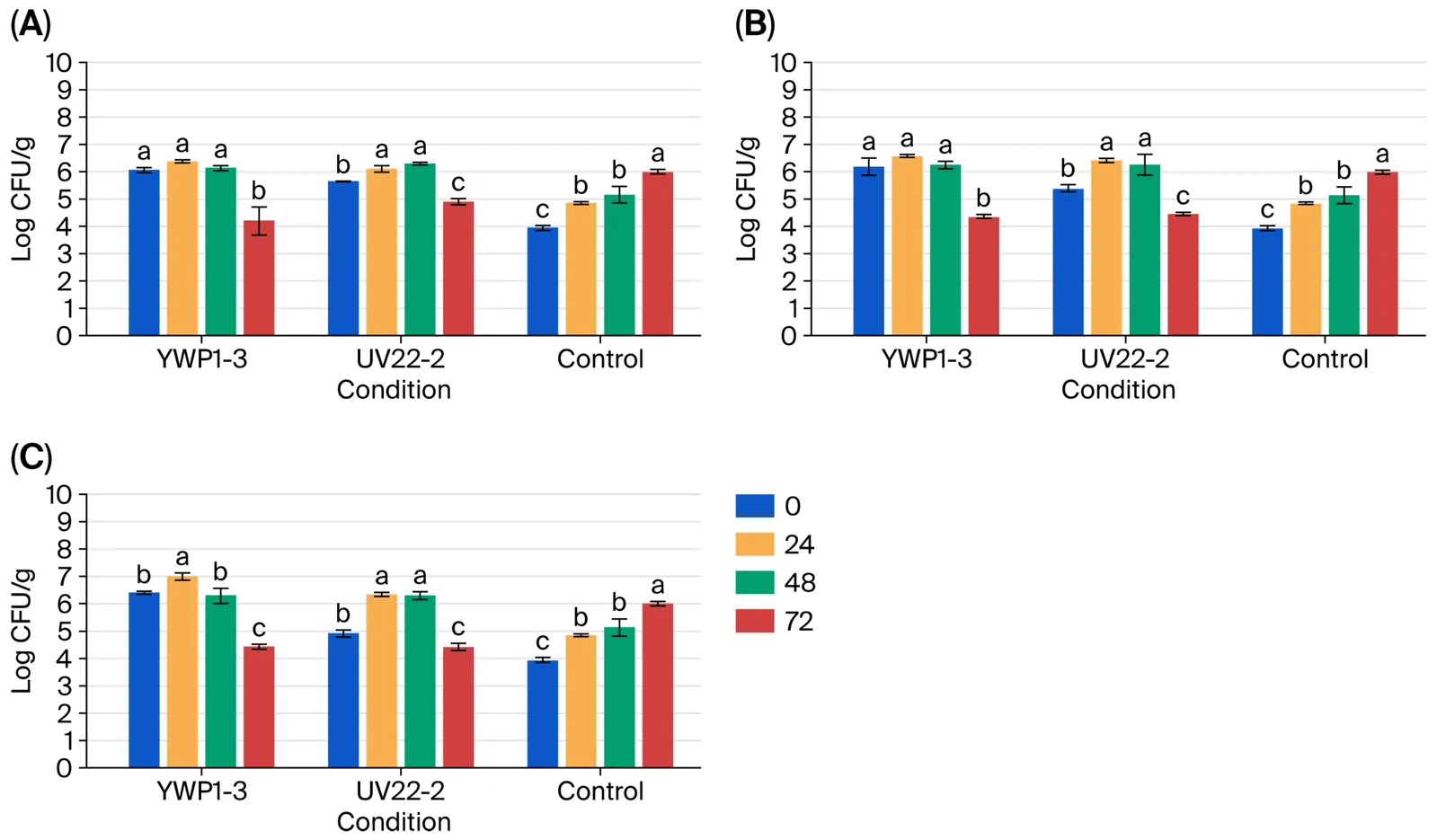
The dynamics of total yeast populations on YMA+ during the fully washed fermentation process at different yeast inoculum concentrations (OD15 (**A**), OD30 (**B**), and OD45 (**C**)). Data represent mean ± standard deviation (*n* = 9). Different letters indicate statistically significant differences among fermentation times at the same starters (*p* < 0.05).

The dynamics of yeast populations during fully washed fermentation showed a characteristic pattern of growth during the early stage of fermentation (24–48 h), followed by a decline toward the final stage (72 h) in the yeast-inoculated samples (Figure 8). In contrast, the control treatment resulted in a continuous increase in the yeast population throughout the fermentation period until 72 h. At an inoculum concentration of OD15, the initial yeast populations under the inoculated treatments (YWP1-3 and UV22-2) ranged from approximately 5.6 to 6.0 log CFU/g, which was clearly higher than that observed in the controls. After 24 h, the yeast populations increased slightly in both inoculated strains and remained at similar levels at 48 h. However, by 72 h, these yeast populations decreased markedly to approximately 4.1–4.9 log CFU/g. In contrast, the control samples showed a continuous increase in yeast population, reaching approximately 6.0 log CFU/g at 72 h. A similar trend was observed at an inoculum concentration of OD30. The yeast populations in the inoculated treatments increased slightly during the first 24 h and remained relatively stable or increased marginally at 48 h before declining markedly at 72 h to approximately 4.3–4.5 log CFU/g. Conversely, the control sample exhibited a gradual increase in yeast populations throughout the fermentation process, reaching approximately 6.0 log CFU/g at the end of fermentation. At an inoculum concentration of OD45, a comparable pattern was observed. The inoculated samples exhibited higher initial yeast populations, which increased slightly during the first 24 h and remained relatively high at 48 h, followed by a significant decline at 72 h. In contrast, the controls showed a steady increase in yeast population until the final stage of fermentation.

#### 3.3.3. Changes in Lactic Acid Bacteria (LAB) Population During Robusta Coffee Fermentation via the Fully Washed Process

**Figure 9:**
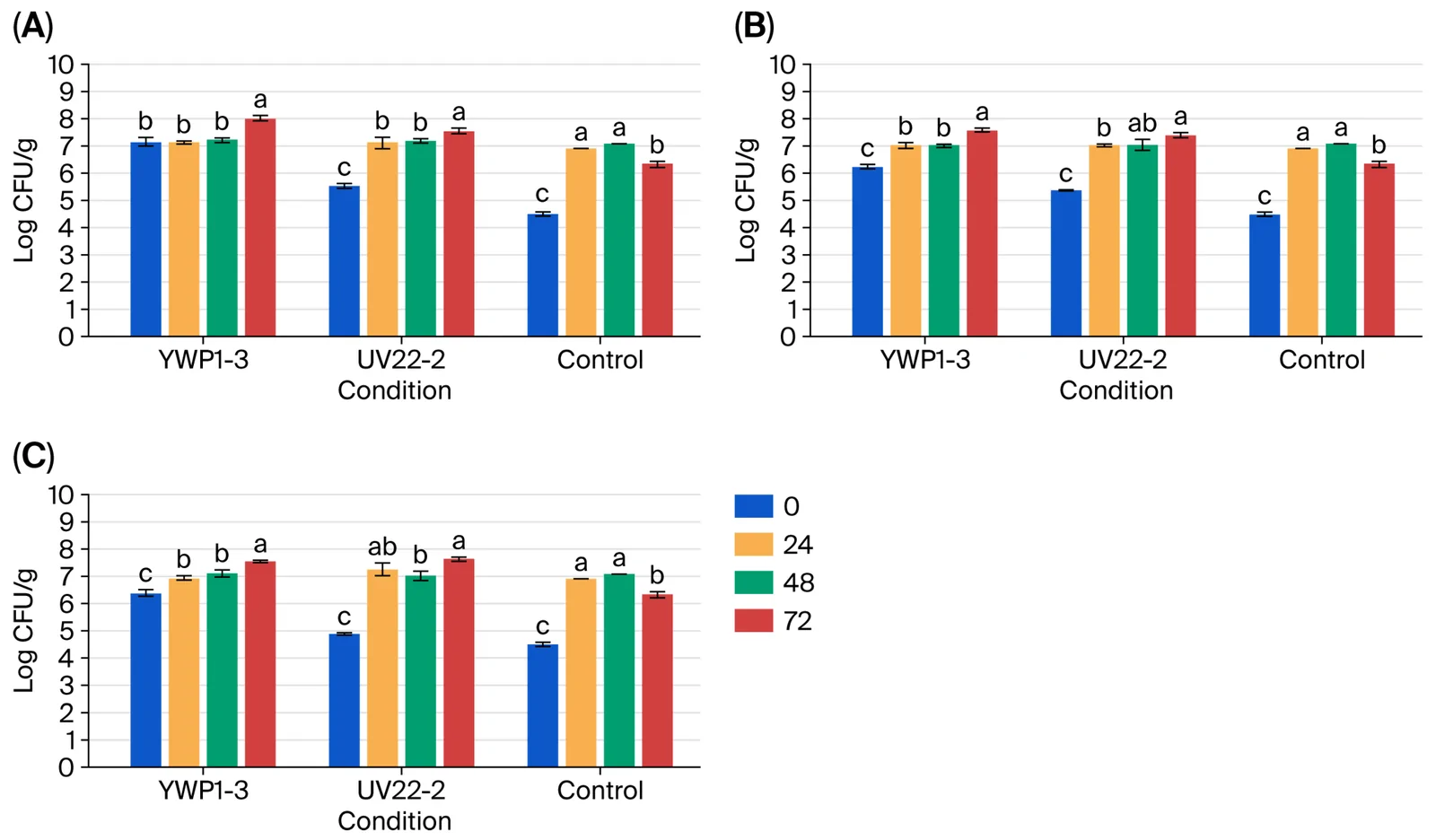
The dynamics of total lactic acid bacterial populations on MRS during fully washed fermentation at different yeast inoculum concentrations (OD15 (**A**), OD30 (**B**), and OD45 (**C**)). Data represent mean ± standard deviation (*n* = 9). Different letters indicate statistically significant differences among fermentation times at the same starters (*p* < 0.05).

The population dynamics of lactic acid bacteria (LAB) during fully washed fermentation showed a consistent trend of increasing throughout the fermentation period, particularly in samples inoculated with yeast (Figure 9). At an inoculum concentration of OD15, the initial LAB populations in the yeast-inoculated samples (YWP1-3 and UV22-2) ranged from approximately 5.5 to 7.0 log CFU/g, which were clearly higher than those observed in the control. After 24 h of fermentation, LAB populations increased following all treatments and continued to rise at 48 h, particularly in the yeast-inoculated groups. By 72 h, LAB populations in the YWP1-3 and UV22-2 treatments increased further, reaching approximately 7.5–8.0 log CFU/g, whereas the control treatment showed a slight decline compared with the population observed at 48 h. A similar trend was observed at an inoculum concentration of OD30. The initial LAB populations ranged from approximately 5.3 to 6.2 log CFU/g and increased markedly during the first 24 h of fermentation. The populations continued to increase or remained relatively stable at 48 h, reaching their highest levels at 72 h in the inoculated samples, with values ranging from approximately 7.3 to 7.6 log CFU/g. In contrast, the controls showed an increase during the first 24–48 h, followed by a slight decline at 72 h. At an inoculum concentration of OD45, a comparable pattern was observed. The initial LAB populations in the inoculated samples were higher than those in the control and increased continuously during the first 24 and 48 h, reaching their maximum levels at 72 h. In contrast, the control samples showed an initial increase during the early fermentation stage, followed by a slight decline toward the end of fermentation. Overall, the fully washed fermentation system resulted in a progressive increase in LAB populations throughout the fermentation period, particularly in the yeast-inoculated samples.

#### 3.3.4. Changes in the Mold Population During Fully Washed Fermentation

**Figure 10:**
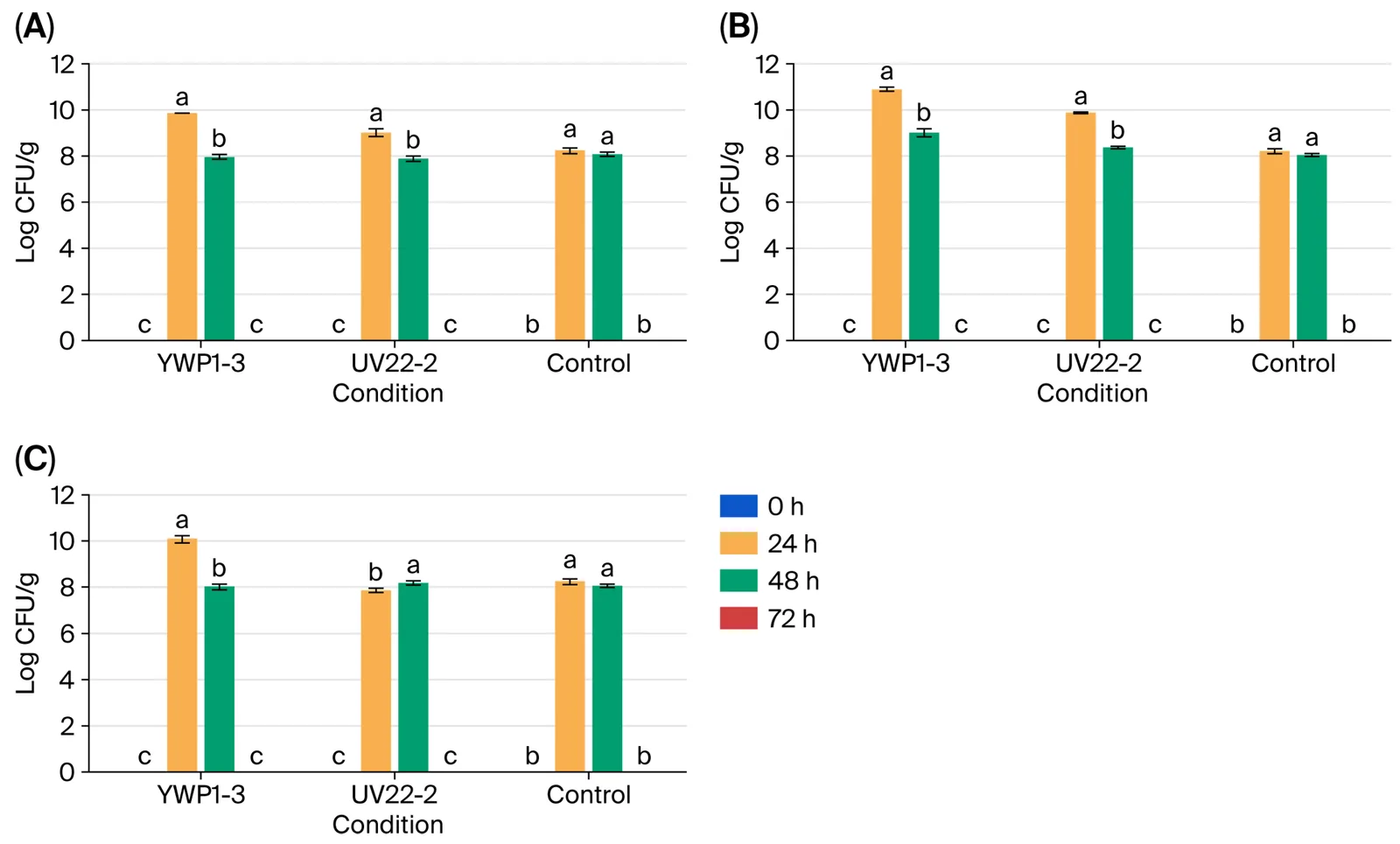
The dynamics of total mold populations on PDA during Robusta coffee fermentation via the fully washed fermentation process at different yeast inoculum concentrations (OD15 (**A**), OD30 (**B**), and OD45 (**C**)). Data represent mean ± standard deviation (*n* = 9). Different letters indicate statistically significant differences among fermentation times at the same starters (*p* < 0.05).

The mold populations showed a rapid increase during the first 24 h, followed by a decrease or stabilization at 48 h. No mold population was detected at 72 h following any treatment, indicating a decline in mold population during the later stage of fermentation (Figure 10). At the OD15 concentration level, no mold was detected at 0 h after any treatment. However, a clear increase in the mold population was observed within the first 24 h. The YWP1-3 treatment resulted in the highest mold population (approximately 9.8 log CFU/g), followed by the UV22-2 and control treatments. At 48 h, the mold population slightly decreased following all treatments but remained at a relatively high level (approximately 7.8–8.0 log CFU/g). At the OD30 concentration level, a similar trend was observed. No mold was detected at 0 h, and the highest population was recorded at 24 h. The YWP1-3-treated samples again showed the highest mold population (approximately 10.8 log CFU/g), followed by the UV22-2 and control samples. At 48 h, the mold population slightly decreased in all treatments but remained above 8.0 log CFU/g. At the OD45 concentration level, a consistent pattern was observed. Mold populations increased rapidly during the first 24 h, with YWP1-3 showing the highest population (approximately 10.0 log CFU/g), followed by a slight decrease at 48 h. Meanwhile, the UV22-2 and control treatments showed similar values and remained relatively stable during the 24–48 h period. Overall, the fully washed fermentation process resulted in rapid mold growth during the early stage of fermentation (within the first 24 h).

### 3.4. Evaluation of Microbial Dynamics During Robusta Coffee Fermentation via the Whole-Cherry Process

#### 3.4.1. Changes in Total Bacterial Populations Determined via PCA During Robusta Coffee Whole-Cherry Fermentation

**Figure 11:**
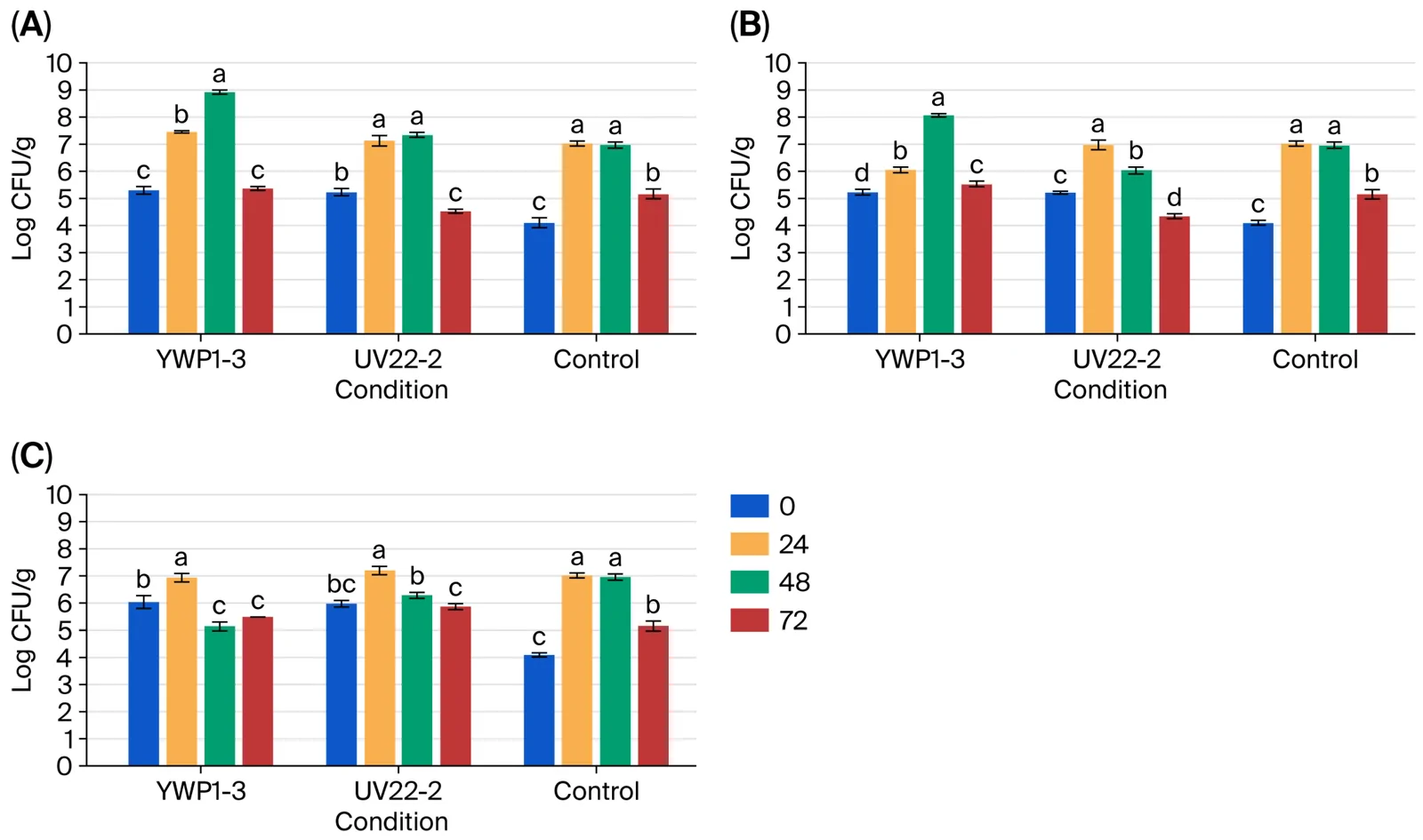
Changes in total bacterial populations determined via PCA during whole-cherry fermentation at different yeast inoculum concentrations (OD15 (**A**), OD30 (**B**), and OD45 (**C**)). Data represent mean ± standard deviation (*n* = 9). Different letters indicate statistically significant differences among fermentation times at the same starters (*p* < 0.05).

Changes in total bacterial populations reflected the dynamics of indigenous bacteria under different fermentation conditions and yeast inoculum concentrations (Figure 11). Overall, all the inoculum levels showed a similar trend: bacterial populations increased during the early-to-middle stages of fermentation (24–48 h) and decreased during the late stage (72 h). At the OD15 concentration level, the initial bacterial population demonstrated a range of approximately 4.0–5.2 log CFU/g at 0 h. Within the first 24 h, the bacterial population increased markedly to around 7.0–7.4 log CFU/g in all treatments. At 48 h, bacterial populations reached their peak levels, particularly in the samples inoculated with the yeast strain YWP1-3, in which the population reached approximately 8.9 log CFU/g, while the populations in the UV22-2-treated and control samples ranged from approximately 6.9 to 7.3 log CFU/g. However, after 72 h of fermentation, the bacterial populations declined significantly in all treatments, decreasing to approximately 4.5–5.3 log CFU/g. This decline indicates the transition to the late stage of fermentation, where environmental conditions become less favorable for bacterial growth. At the OD30 concentration level, a similar trend was observed compared with OD15. The initial bacterial population ranged from approximately 4.0 to 5.2 log CFU/g and increased during the first 24 h of fermentation. At 48 h, the bacterial populations increased markedly and reached the highest levels observed in this experiment, ranging from approximately 6.0 to 8.0 log CFU/g in some treated samples. After 72 h, bacterial populations decreased to approximately 4.3–5.5 log CFU/g. This reduction may reflect environmental changes within the fermentation system, such as the accumulation of organic acids and the depletion of readily available nutrients. At the OD45 concentration level, the initial bacterial population was relatively high, approximately 4.0–6.0 log CFU/g, and slightly increased during the first 24 h to about 6.9–7.2 log CFU/g. However, at 48 h, the increase in bacterial population was less pronounced than that observed at OD15 and OD30, with values ranging from approximately 5.0 to 6.9 log CFU/g. At 72 h, the bacterial population ranged from approximately 5.2 to 5.9 log CFU/g, showing less fluctuation compared with the lower inoculum concentrations. Overall, the results indicate that indigenous bacterial populations in the whole-cherry fermentation system increased markedly during the first 24–48 h before declining at 72 h across all yeast inoculum levels. The OD15 and OD30 concentrations appeared to promote greater bacterial proliferation during the middle stage of fermentation, whereas the OD45 level may partially limit bacterial growth.

#### 3.4.2. Population Dynamics of Yeasts During Whole-Cherry Fermentation of Robusta Coffee

**Figure 12:**
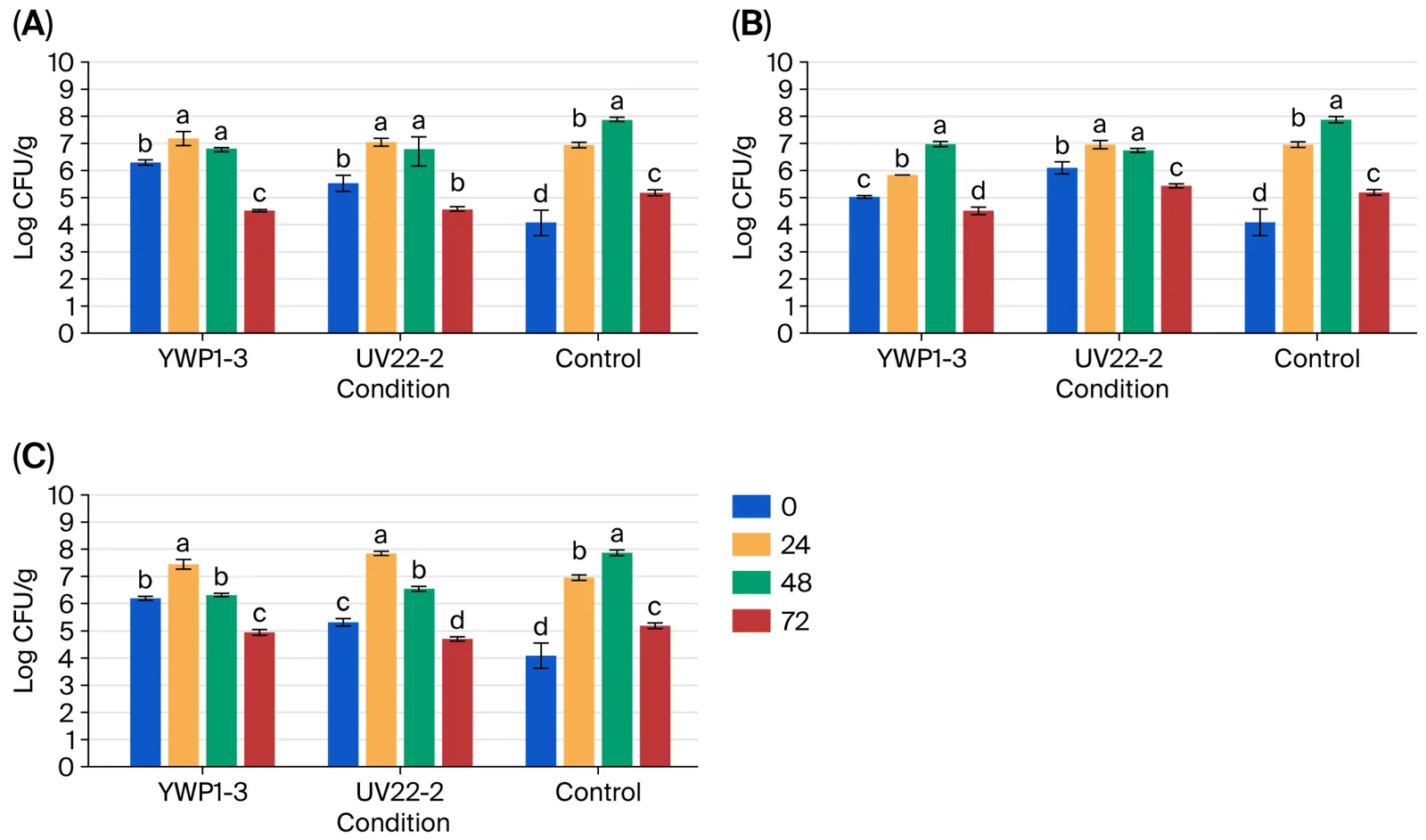
Population dynamics of yeasts on YMA+ during whole-cherry fermentation of robusta coffee at different yeast inoculum concentrations (OD15 (**A**), OD30 (**B**), and OD45 (**C**)). Data represent mean ± standard deviation (*n* = 9). Different letters indicate statistically significant differences among fermentation times at the same starters (*p* < 0.05).

The changes in total yeast populations determined via YMA+ reflected the abundance of both the inoculated yeasts and indigenous yeasts present in the fermentation system of Robusta coffee from Loei Province (Figure 12). Overall, at all inoculum concentrations (OD15, OD30, and OD45), yeast populations increased markedly during the first 24–48 h of fermentation before declining at 72 h. At the OD15 concentration level, the initial yeast population ranged from approximately 4.1 to 6.3 log CFU/g at 0 h, with the inoculated samples showing higher initial values than the controls. During the first 24 h, yeast populations increased significantly, reaching approximately 6.9–7.2 log CFU/g. The populations remained relatively high at 48 h, with some treated samples showing values of around 6.8–7.8 log CFU/g. However, by 72 h, the yeast population decreased to approximately 4.5–5.2 log CFU/g, indicating the transition to the late stage of fermentation, when environmental conditions become less favorable for yeast survival. At the OD30 concentration level, a similar increasing trend was observed. The initial yeast population ranged from approximately 4.1 to 6.1 log CFU/g and increased during the first 24 h to approximately 5.8–7.0 log CFU/g. At 48 h, some treated samples reached peak values of approximately 7.0–7.9 log CFU/g, particularly in the control and for certain treatments, which showed slightly higher peaks than those observed at OD15. After 72 h, the yeast population declined to approximately 4.5–5.4 log CFU/g, reflecting nutrient depletion and environmental changes within the fermentation system. At the OD45 concentration level, the initial yeast population was relatively high (approximately 4.1–6.2 log CFU/g) and increased markedly during the first 24 h, with populations in some treated samples reaching approximately 7.4–7.9 log CFU/g. However, at 48 h, yeast populations did not increase substantially beyond those observed at OD30 and tended to remain stable or increase only slightly. By 72 h, yeast populations decreased to approximately 4.7–5.2 log CFU/g. Overall, across all three inoculum levels, yeast populations reached their highest levels during the 24–48 h period, corresponding to the active fermentation phase of the whole-cherry process. The OD30 and OD45 treatments tended to produce slightly higher maximum yeast populations than OD15. However, the difference between OD30 and OD45 was not pronounced. At 72 h, all treated samples exhibited a similar decline in yeast populations, indicating the transition to the late fermentation stage. These results suggest that yeast inoculation in the fermentation process promotes rapid yeast proliferation during the early stage of fermentation and maintains high population levels during the 24–48 h period before declining at 72 h.

#### 3.4.3. Changes in the Population of Lactic Acid Bacteria (LABs)

**Figure 13:**
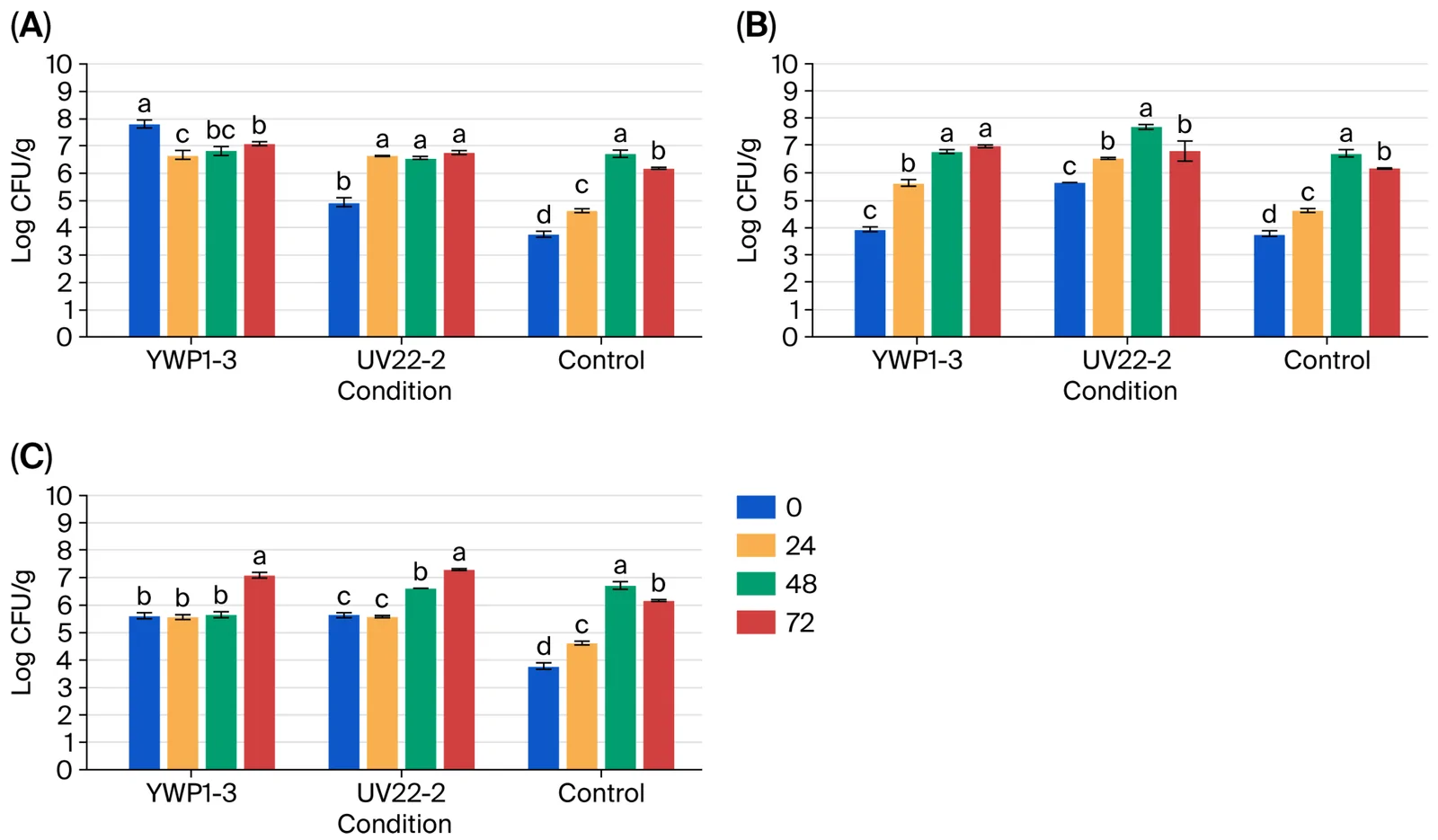
Changes in the population of lactic acid bacteria (LABs) on MRS during whole-cherry fermentation of Robusta coffee at different yeast inoculum concentrations (OD15 (**A**), OD30 (**B**), and OD45 (**C**)). Data represent mean ± standard deviation (*n* = 9). Different letters indicate statistically significant differences among fermentation times at the same starters (*p* < 0.05).

Changes in the population of lactic acid bacteria (LABs), determined using MRS medium, reflected the dynamics of indigenous LABs present in the Robusta coffee from Loei Province during whole-cherry fermentation (Figure 13). Overall, LAB populations tended to increase in the middle-to-late stages of fermentation, with different patterns observed depending on the concentration of the yeast inoculum. At the OD15 inoculum level, the LAB population in the sample inoculated with YWP1-3 initially appeared relatively high (approximately 7.8 log CFU/g) and slightly decreased during the first 24–48 h before increasing again at 72 h to around 7.0 log CFU/g. In contrast, the UV22-2-treated sample started at a lower level (of approximately 5.0 log CFU/g) but gradually increased throughout the fermentation period (24–72 h), reaching approximately 6.7–6.8 log CFU/g. The control treatment exhibited the lowest initial LAB population (approximately 3.8 log CFU/g); however, it increased markedly at 48 h (to approximately 6.7 log CFU/g) before declining slightly at 72 h. At the OD45 inoculum level, LAB populations showed a clearer increase during the later stage of fermentation. The YWP1-3- and UV22-2-treated samples showed initial values of approximately 5.6 log CFU/g and increased continuously until 72 h, reaching approximately 7.0 log CFU/g. In the control samples, although the initial population was lower (approximately 3.8 log CFU/g), a substantial increase was observed at 48 h (approximately 6.7 log CFU/g), and the population remained relatively high at 72 h. Overall, LAB populations clearly increased during the 48–72 h fermentation period, corresponding to the stage at which organic acids accumulated and pH values decreased. This pattern reflects the important role of LABs during the middle-to-late stages of the fermentation process. In addition, the higher yeast inoculum concentration (OD45) resulted in a slightly greater increase in LAB populations during the late fermentation stage compared with OD15.

#### 3.4.4. Changes in Mold Populations During Whole-Cherry Fermentation of Robusta Coffee

**Figure 14:**
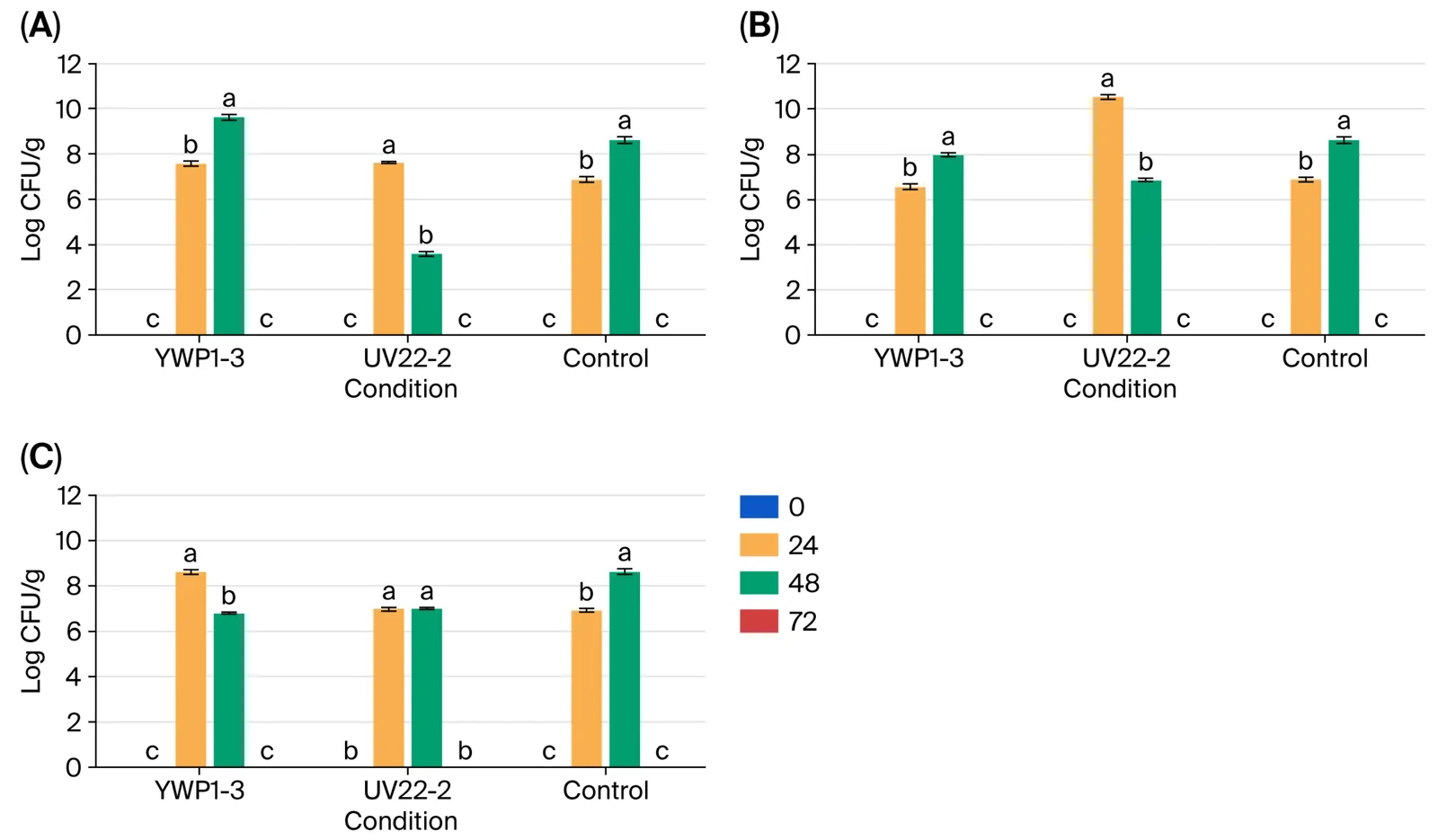
The dynamics of total mold populations on PDA during whole-cherry fermentation of Robusta coffee at different yeast inoculum concentrations (OD15 (**A**), OD30 (**B**), and OD45 (**C**)). Data represent mean ± standard deviation (*n* = 9). Different letters indicate statistically significant differences among fermentation times at the same starters (*p* < 0.05).

The changes in mold populations determined using the PDA medium revealed the dynamics of molds under whole-cherry fermentation (Figure 14). In general, molds showed a tendency to increase during the early to mid-stages of fermentation (24–48 h) but did not exhibit a clear increase in the later stages of fermentation. The pattern of change varied depending on the concentration of the yeast inoculum. At the OD15 inoculum level, molds were not detected at the initial time point (0 h). However, over the first 24 h, mold populations increased in all treated samples, reaching approximately 6.9–7.6 log CFU/g. At 48 h, the sample inoculated with the yeast strain YWP1-3 showed the highest increase (approximately 9.6 log CFU/g). In contrast, the UV22-2-treated samples showed a decrease compared with the 24 h value (approximately 3.5 log CFU/g), while the control value increased to approximately 8.6 log CFU/g. No mold was detected at 72 h, which may reflect a decline in mold populations during the late stage of fermentation. At the OD30 inoculum level, a clear increase in mold populations was observed at 24 h, with some treated samples reaching approximately 6.5–10.5 log CFU/g, particularly the UV22-2 samples, which showed relatively high values at the early stage. At 48 h, mold populations generally remained large (approximately 6.8–8.6 log CFU/g), with the control and some treated samples showing a slight increase. However, mold was not detected at 72 h, indicating that the molds were unable to persist or further proliferate during the late fermentation stage. At the OD45 inoculum level, mold populations increased during the first 24 h (approximately 6.8–8.6 log CFU/g). In some samples, a slight increase was observed at 48 h (approximately 6.8–8.6 log CFU/g), particularly in the control, which showed the highest values. However, similar to OD15 and OD30, no increase in mold populations was observed at 72 h.

### 3.5. Sensory Analysis

The sensory evaluation results showed differences in cup quality associated with the fermentation process, yeast strain, inoculum concentration, and fermentation duration (Table 2 and Table 3). In the whole-cherry samples, the highest final cup score was obtained for the YWP1-3 treatment at an inoculum concentration of OD_600_ = 45 after 72 h of fermentation (82.00 ± 0.15 points). This coffee was characterized by chocolate, raisin, and sweet notes, together with a round cup profile and a long aftertaste. The uninoculated control coffee fermented for 24 h received the second-highest score (81.00 ± 0.50 points) and was characterized by chocolate, hazelnut, dried-fruit sweetness, a well-structured cup, and a dry mouthfeel upon cooling. Overall, the whole-cherry treatments produced cup profiles commonly characterized by chocolate, cocoa, nutty, grain-like, and sweet notes. The favorable performance of YWP1-3 at OD_600_ = 45 after 72 h suggests that this particular combination of strain, inoculum concentration, and fermentation duration may enhance the sensory quality of whole-cherry-processed Robusta coffee. Nevertheless, this response was treatment-specific, as increasing either the inoculum concentration or fermentation time did not consistently improve the cup score across all treatments. Some samples fermented for 48 h were described as flat, watery, low in acidity, or bitter and received comparatively lower scores. The whole-cherry samples inoculated with UV22-2 received scores ranging from 72.75 to 77.50 points and were generally characterized by roasted, grainy, hay-like, papery, or bland notes, indicating lower sensory complexity than the highest-scoring YWP1-3 treatment (Table 2). Among fully washed samples, final cup scores ranged from 64.50 to 76.25 points and were generally lower and more variable than those recorded for the whole-cherry process samples. The highest score was obtained with UV22-2 at OD_600_ = 45 after 24 h of fermentation (76.25 ± 0.84 points), with the sample characterized by chocolate, banana, corn, savory notes, and a medium body. The lowest score was observed for YWP1-3 at OD_600_ = 15 after 72 h of fermentation (64.50 ± 0.14 points); this sample exhibited grainy and corn-like notes, a phenolic defect, harshness, blackish character, and a bold, powdery mouthfeel. Several samples produced via fully washed fermentation particularly with extended fermentation times, exhibited undesirable sensory characteristics, including phenolic, rubbery, earthy, harsh, bitter, astringent, and sandy attributes. However, the effects of prolonged fermentation were not uniform across all treatments. For example, UV22-2 at OD_600_ = 30 and 45 after 72 h yielded scores of 75.50 ± 0.68 and 74.25 ± 0.67 points, respectively. Therefore, the development of undesirable characteristics appeared to depend on the interaction among yeast strain, inoculum concentration, and fermentation duration rather than fermentation time alone (Table 3). Descriptively, the whole-cherry process produced higher final cup scores, ranging from 72.00 to 82.00 points, than the fully washed process, for which scores ranged from 64.50 to 76.25 points. The whole-cherry samples also tended to exhibit sweeter, smoother, and more rounded sensory profiles. In particular, the sample that underwent YWP1-3 at OD_600_ = 45 after 72 h showed promising sensory performance. By contrast, the fully washed process appeared to be more likely to result in undesirable sensory characteristics under some extended-fermentation conditions. These findings indicate that the optimum fermentation duration should be selected specifically for each combination of processing method, yeast strain, and inoculum concentration.

## 4. Discussion

### 4.1. Safety Considerations and Implications for Industrial Application

The safety assessment of microbial starter cultures is an important component of their evaluation for use in food fermentation. Although selected yeasts may improve fermentation control and contribute to desirable sensory characteristics, their application requires assessment of potential strain-specific safety concerns. In the present study, *Wickerhamomyces anomalus* YWP1-3 and UV22-2 showed no hemolytic activity and no detectable cytotoxic effects toward Caco-2 intestinal epithelial cells at the lower concentrations tested. Cytotoxic effects were observed only at higher concentrations of the fermentation supernatants, indicating a concentration-dependent response. However, because actual exposure following coffee processing, roasting, and brewing was not evaluated, the relevance of these findings to consumer exposure cannot be directly established. The MTT assay showed a concentration-dependent reduction in Caco-2 cell viability following exposure to fermentation supernatants from both YWP1-3 and UV22-2 (Figure 1 and Figure 2). Viability generally remained relatively high at concentrations of ≤12.5% (*v*/*v*), whereas larger reductions were observed at 25% (*v*/*v*) and above. Changes associated with increasing sample concentration were more apparent than changes across the 0–72 h fermentation period. At moderate concentrations, the UV22-2 samples appeared to produce a smaller reduction in viability than the YWP1-3 samples. The reduction in viability at higher concentrations cannot be attributed exclusively to compounds produced by either yeast strain. In particular, the decrease observed with the YWP1-3 supernatant at these concentrations was not different from that observed with the corresponding uninoculated fermentation-matrix control. Fermentation and dilution of coffee mucilage may alter pH, osmolality, organic acid concentrations, and other matrix properties that can affect Caco-2 cell metabolism and the MTT signal. These findings provide preliminary information on the responses of Caco-2 cells to diluted fermentation supernatants under the tested conditions.

The antifungal susceptibility assay provided preliminary MIC profiles for both strains under the experimental conditions. Nevertheless, because species-specific clinical breakpoints and epidemiological cutoff values are unavailable for *W. anomalus* against the tested antifungal agents, the strains could not be classified as susceptible or resistant. Therefore, the findings should be interpreted as descriptive preliminary safety data rather than definitive evidence of antifungal susceptibility or food safety. The absence of hemolytic activity, together with the observed cytotoxicity and antifungal susceptibility profiles, was generally consistent with the safety considerations recommended for microorganisms intended for use in food systems [13,14].

According to the Thai list of controlled biological agents, *Wickerhamomyces anomalus* is classified as a Risk Group 1 microorganism under code F-1-0242. This classification indicates that the species is generally considered unlikely to cause disease in healthy humans under appropriate handling conditions. The absence of hemolytic activity and the preliminary cytotoxicity and antifungal susceptibility profiles observed for strains YWP1-3 and UV22-2 were generally consistent with this low-risk classification, based on the safety endpoints examined in the present study. However, classification of the species as a Risk Group 1 microorganism does not constitute regulatory approval of these individual strains for food use or confirm their safety under all processing and consumption conditions. Therefore, additional strain-level assessments, including genomic screening for virulence and acquired antifungal-resistance determinants, evaluation under realistic processing and exposure conditions, antimicrobial resistance genes, the production of undesirable metabolites, genomic characterization, and toxin-producing potential are required before their food-grade status and suitability for industrial-scale application can be conclusively established.

### 4.2. Yeast-Driven Mucilage Degradation and Its Impact on Fermentation Biochemistry

The present study demonstrates that controlled yeast inoculation influenced the biochemical trajectory of Robusta coffee fermentation, with effects varying between processing systems. In the modified whole-cherry process, °Brix generally decreased during the first 24 h, followed by treatment-dependent fluctuations during the later stages of fermentation. In contrast, the fully washed process showed an increase in °Brix at 72 h in the yeast-inoculated samples. These changes may reflect the combined effects of microbial substrate utilization, the release of soluble compounds from the coffee matrix, and sampling variability.

Coffee mucilage is composed primarily of pectins, cellulose, and hemicellulose, which can serve as substrates for microbial metabolism during fermentation. Previous studies have reported that yeasts associated with coffee fermentation, including *Wickerhamomyces anomalus*, may produce extracellular enzymes, such as pectinases and glycosidases, that facilitate mucilage degradation [12,18,19,20]. Therefore, the treatment-dependent changes in °Brix observed in the inoculated samples may partly reflect differences in microbial activity and the release or utilization of soluble compounds. However, individual sugars, pectin degradation, and extracellular enzyme activities were not directly measured in the present study. Consequently, the observed °Brix patterns cannot be conclusively attributed to enhanced enzymatic degradation of mucilage. Further studies incorporating sugar profiling, pectin analysis, and enzyme-activity measurements are required to clarify the mechanisms underlying these changes.

### 4.3. Coupling Between Sugar Release and Acidification Dynamics

The evolution of pH during fermentation revealed a dynamic interplay between substrate utilization and metabolite production. The slight increase in pH during the early stage of fermentation has been reported in previous studies and may be attributed to microbial metabolism of amino acids and buffering compounds [19,21]. This was followed by a pronounced decrease in pH at later stages, reflecting the accumulation of organic acids such as lactic, acetic, and succinic acids produced by both yeasts and associated bacterial communities [22,23].

Importantly, in the yeast-inoculated fully washed samples, the late-stage increase in °Brix occurred alongside a decrease in pH. This pattern may reflect changes in soluble compounds and acidification during fermentation; however, °Brix does not identify individual sugars or establish their source. Because carbohydrate composition, pectin degradation, and extracellular enzyme activities were not measured, the processes underlying these concurrent changes remain uncertain. Similar fermentation dynamics have been reported in controlled coffee fermentations where yeast-driven mucilage degradation provides substrates for subsequent microbial metabolism [6,12,19]. Such coupling of biochemical processes has been identified as a key driver of flavor precursor formation in coffee fermentation [6]. The more consistent acidification pattern observed in the whole-cherry process further indicates a more controlled fermentation environment, which may contribute to improved flavor stability and reproducibility.

### 4.4. Strain-Dependent Effects on Fermentation Performance

Both yeast strains (YWP1-3 and UV22-2) produced comparable °Brix levels at higher inoculum concentrations (OD30 and OD45), suggesting similar capacities for mucilage degradation during the later fermentation stages. However, the slightly higher °Brix values observed for YWP1-3 at the lower inoculum concentration (OD15) may indicate differences in metabolic activity or extracellular enzyme production between the strains. Strain-dependent variability in enzymatic capacity and metabolic pathways has been widely reported in yeast-driven coffee fermentation and can significantly influence fermentation kinetics and metabolite production [19,20]. From a technological perspective, these results highlight the importance of controlled yeast inoculation in modulating the biochemical environment of coffee fermentation. Enhanced mucilage degradation and gradual acidification may influence the formation of volatile precursors and flavor-active metabolites that ultimately contribute to coffee aroma and cup quality [6,21].

### 4.5. Microbial Dynamics During Fermentation

#### 4.5.1. Microbial Population Dynamic and Environmental Shifts

Microbial population dynamics during coffee fermentation is a complex ecological process influenced by substrate availability, environmental conditions, and microbial interactions. In this study, controlled yeast inoculation was clearly demonstrated to influence the dynamics of indigenous microbial populations, particularly bacteria and lactic acid bacteria (LABs). Higher total culturable yeast counts were observed in some inoculated treatments during early fermentation. However, because YMA+ enumeration did not distinguish the inoculated strains from indigenous yeasts, strain-specific dominance could not be established. This observation is consistent with previous findings that starter cultures can act as ecological modulators, shaping the fermentation microbiome and promoting more predictable fermentation outcomes [10,19]. The increase in LAB populations during the mid-to-late stages of fermentation further reflects the transition toward an acidified environment, in which LABs become more competitive. Such dynamic patterns are critical for the development of desirable flavor compounds and overall coffee quality [6]. Moreover, the observed differences between strains YWP1-3 and UV22-2 suggest strain-specific metabolic capacities, likely related to variations in enzyme production and substrate utilization. These findings highlight the importance of strain selection in designing targeted fermentation strategies for coffee processing.

#### 4.5.2. Effect of Yeast Inoculation on Mold Growth

Interestingly, higher yeast inoculum concentrations did not result in increased mold populations. Instead, mold growth was largely confined to the early- to mid-fermentation stages and tended to decline during later stages. This observation suggests that inoculated yeasts may exert a competitive effect against filamentous fungi through rapid substrate utilization, environmental modification, or the production of inhibitory metabolites. Several yeast species associated with coffee fermentation have been reported to exhibit antagonistic activity against undesirable microorganisms [6,22]. Rapid colonization by inoculated yeasts may therefore limit ecological niches available for mold proliferation and contribute to microbial stabilization during fermentation. Such microbial interactions are increasingly recognized as key mechanisms underlying controlled fermentation processes and microbial ecosystem stability [19].

### 4.6. Sensory Implications of Fermentation Conditions

#### 4.6.1. Influence of Fermentation Parameters on Cup Quality

The sensory evaluation demonstrated that fermentation process, yeast strain, inoculum concentration, and fermentation duration collectively influenced the cup quality of Robusta coffee. The whole-cherry fermentation system generally produced higher sensory scores and more balanced cup profiles compared with the fully washed process. This finding highlights the importance of fermentation conditions in shaping the biochemical transformations occurring in coffee mucilage and the formation of flavor precursors responsible for coffee aroma and flavor development. Coffee fermentation is a complex microbe-driven process in which yeasts, bacteria, and filamentous fungi metabolize sugars, pectins, and other organic compounds present in the mucilage, producing metabolites such as alcohols, organic acids, esters, and aldehydes that contribute to coffee flavor complexity [6,19]. Controlled microbial fermentation has been reported to enhance sensory attributes such as sweetness, fruitiness, and body by promoting the formation of aroma-active compounds [20].

#### 4.6.2. Sensory Characteristics Resulting from Whole-Cherry Fermentation

Following whole-cherry fermentation, the highest sensory score was obtained with YWP1-3 at OD_600_ = 45 after 72 h, followed by the uninoculated control fermented for 24 h. A comparable score was also observed in the samples inoculated with strain YWP1-3 at OD45 for 72 h, suggesting that more yeast inoculum combined with extended fermentation may promote the formation of flavor-active metabolites. Yeasts can convert mucilage-derived sugars into alcohols, esters, and other aromatic compounds that enhance sweetness and flavor complexity [19,20]. The dominance of chocolate, cocoa, nutty, and sweet flavor notes in several whole-cherry samples may therefore be associated with yeast-mediated production of aromatic precursors that later contribute to desirable roasting-derived compounds.

#### 4.6.3. Sensory Defects Associated with Prolonged Fermentation

Prolonged fermentation via the fully washed process frequently produced undesirable sensory attributes, such as phenolic, earthy, rubbery, or astringent notes, particularly at 72 h. These defects are commonly associated with excessive microbial metabolism and the accumulation of organic acids or other secondary metabolites that negatively affect flavor quality [6,19]. Prolonged fermentation may also promote the activity of indigenous microorganisms capable of producing off-flavor compounds [24]. Interestingly, a fully washed fermentation duration of approximately 24 h generally produced better cup quality, suggesting that shorter fermentation times may help maintain a balance between mucilage degradation and flavor precursor formation while preventing excessive microbial metabolism.

### 4.7. Implications for Controlled Coffee Fermentation: Advantages of Yeast Starter Cultures

Although spontaneous fermentation occasionally produced high sensory scores, it cannot guarantee consistent coffee quality because the microbial community composition may vary depending on environmental conditions such as temperature, humidity, fruit maturity, and processing hygiene [6,19]. Such variability can lead to inconsistent fermentation performance and unpredictable sensory outcomes. The use of defined yeast starter cultures offers a promising strategy to improve fermentation consistency. Yeasts may rapidly influence the fermentation environment and dynamics of culturable microbial populations during the early stages of fermentation and suppress undesirable microorganisms [6,23]. The starter treatments were associated with treatment-dependent changes in culturable microbial populations, physicochemical parameters, and sensory characteristics under the conditions tested.

### 4.8. Relationship Between Physicochemical Parameters, Microbial Populations, and Sensory Quality

Correlation analysis was performed separately for the fully washed and modified whole-cherry processes to investigate the relationships among physicochemical parameters, culturable microbial populations, and total cupping scores. Distinct correlation patterns were observed between the two processes, indicating that the relationships between fermentation variables and sensory quality were process-dependent. Similar effects of postharvest processing on coffee microbial ecology, metabolite formation, and sensory characteristics have been reported previously [10,19,25].

#### 4.8.1. Fully Washed Process

**Figure 15:**
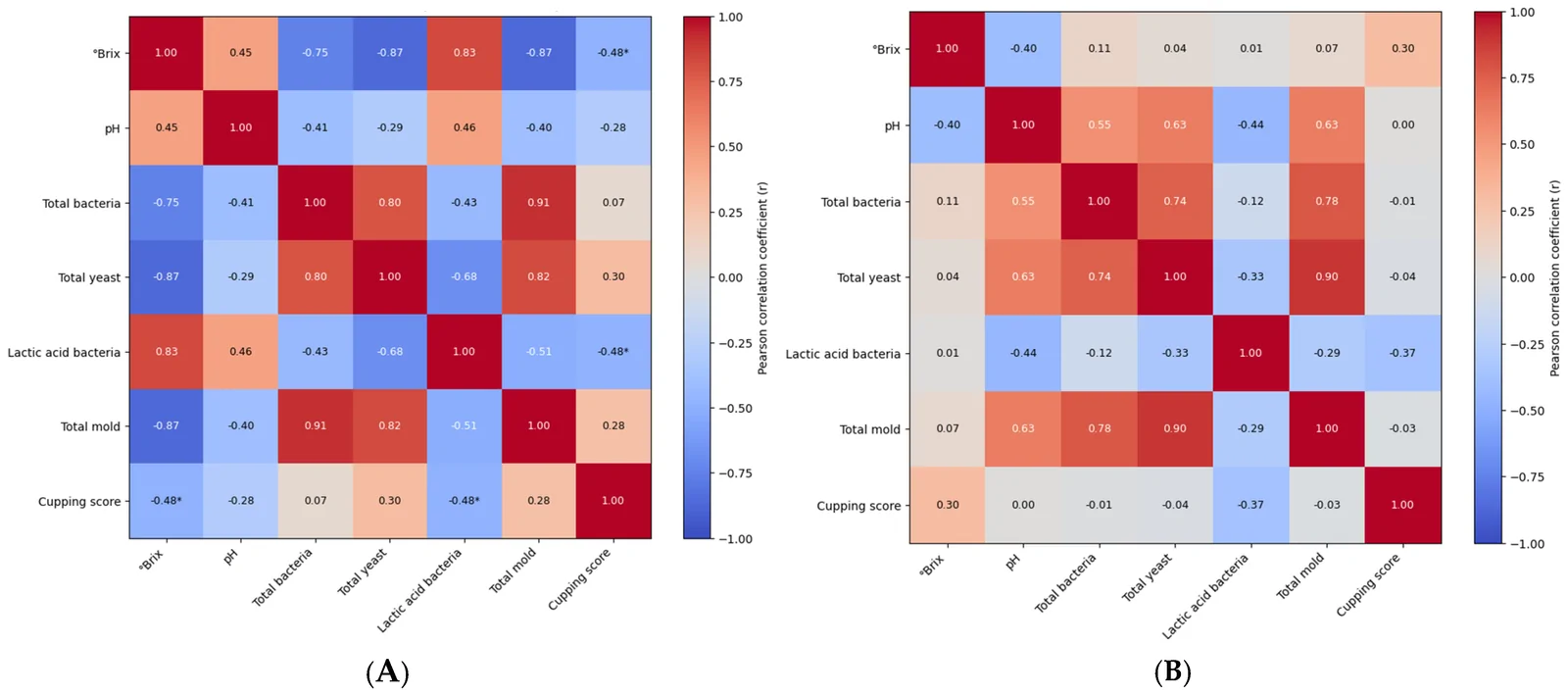
Pearson correlation heatmaps showing the relationships among physicochemical parameters (°Brix and pH), culturable microbial populations (total bacteria, total yeast, lactic acid bacteria, and total mold), and cupping score of Robusta coffee fermented using (**A**) the Fully Washed process and (**B**) the modified whole-cherry process. Correlation coefficients (r) are displayed within each cell.

In the fully washed process, the total cupping score was moderately and negatively correlated with °Brix (r = −0.48, *p* < 0.05) and culturable lactic acid bacteria (r = −0.48, *p* < 0.05). Correlations with pH (r = −0.28), total bacteria (r = 0.07), total yeast (r = 0.30), and total mold (r = 0.28) were weaker (Figure 15A).

The negative relationship between °Brix and cupping score may reflect microbial utilization of soluble substrates during fermentation rather than a direct reduction in perceived sweetness. During coffee fermentation, microorganisms metabolize mucilage carbohydrates and produce organic acids, alcohols, and volatile compounds that can influence sensory characteristics [19,26]. This interpretation is supported by the strong negative correlations of °Brix with total bacteria (r = −0.75), total yeast (r = −0.87), and total mold (r = −0.87), suggesting that increasing microbial populations occurred concurrently with changes in soluble solids.

The negative association between lactic acid bacteria and cupping score should also be interpreted cautiously. Previous studies have demonstrated that specific lactic acid bacteria can improve coffee sensory quality [27]. However, the present MRS counts represent total culturable lactic acid bacteria rather than individual species or strains. Therefore, microbial abundance alone may not reflect the production of desirable flavor metabolites. Similarly, the weak relationship between pH and cupping score indicates that fermentation pH alone does not adequately predict sensory quality.

#### 4.8.2. Modified Whole-Cherry Process

In the modified whole-cherry process, none of the physicochemical or microbial variables showed a significant correlation with total cupping score. °Brix showed a weak-to-moderate positive association (r = 0.30), whereas pH (r ≈ 0.00), total bacteria (r = −0.01), total yeast (r = −0.04), and total mold (r = −0.03) showed little relationship with cupping score. Lactic acid bacteria showed a moderate negative trend (r = −0.37), but this relationship was not statistically significant (*p* > 0.05) (Figure 15B).

Nevertheless, strong positive correlations occurred among microbial populations, particularly between total yeast and total mold (r = 0.90), total bacteria and total mold (r = 0.78), and total bacteria and total yeast (r = 0.74). These findings suggest coordinated changes in culturable microbial populations during modified whole-cherry fermentation, although population size alone did not explain final sensory quality. The intact skin, pulp, and mucilage surrounding the beans may create a more complex fermentation environment in which substrate diffusion, microbial interactions, and metabolite production collectively determine sensory outcomes [19,25].

Overall, the contrasting correlation patterns demonstrate that relationships among physicochemical parameters, microbial populations, and sensory quality depend on the processing method. These correlations indicate associations rather than causality. Moreover, °Brix and pH should not be interpreted as direct measures of perceived sweetness and acidity, respectively; treatment-matched sensory attribute scores would be required to establish these specific physicochemical–sensory relationships.

## 5. Conclusions

This study extends previous work on *Wickerhamomyces anomalus* YWP1-3 and UV22-2 by evaluating their application in *Coffea canephora* fermentation across fully washed and modified whole-cherry processes, alongside preliminary biosafety and sensory assessments. Neither strain showed hemolytic activity under the conditions tested. The antifungal assays yielded descriptive MIC values, which do not support classification of the strains as susceptible or resistant. Microbial population dynamics and sensory outcomes varied by processing method, strain, inoculum level, and fermentation time. Following the modified whole-cherry process, selected inoculated samples showed favorable sensory attributes, including sweetness and a rounder mouthfeel, although the highest-scoring sample overall was the uninoculated 24 h control. These findings provide process-specific evidence on the behavior of the two yeast strains during Robusta coffee fermentation and identify conditions for further investigation.

## Figures and Tables

**Table 1:** The fermentation conditions for coffee processing were systematically designed for both the fully washed and modified whole-cherry fermentation processes using selected microbial starter cultures to evaluate their effects on fermentation dynamics.

Treatment Name *	Yeast Strains	Fermentation Process	Starter Concentration (OD 600 nm)	Incubation Time (h)
Control WC (24 h)	Without inoculation	Whole-cherry	-	24
Control WC (48 h)	Without inoculation	Whole-cherry	-	48
Control WC (72 h)	Without inoculation	Whole-cherry	-	72
1-3 15 WC (24 h)	*W. anomalus* YWP1-3	Whole-cherry	15	24
1-3 15 WC (48 h)	*W. anomalus* YWP1-3	Whole-cherry	15	48
1-3 15 WC (72 h)	*W. anomalus* YWP1-3	Whole-cherry	15	72
1-3 30 WC (24 h)	*W. anomalus* YWP1-3	Whole-cherry	30	24
1-3 30 WC (48 h)	*W. anomalus* YWP1-3	Whole-cherry	30	48
1-3 30 WC (72 h)	*W. anomalus* YWP1-3	Whole-cherry	30	72
1-3 45 WC (24 h)	*W. anomalus* YWP1-3	Whole-cherry	45	24
1-3 45 WC (48 h)	*W. anomalus* YWP1-3	Whole-cherry	45	48
1-3 45 WC (72 h)	*W. anomalus* YWP1-3	Whole-cherry	45	72
22-2 15 WC (24 h)	*W. anomalus* UV22-2	Whole-cherry	15	24
22-2 15 WC (48 h)	*W. anomalus* UV22-2	Whole-cherry	15	48
22-2 15 WC (72 h)	*W. anomalus* UV22-2	Whole-cherry	15	72
22-2 30 WC (24 h)	*W. anomalus* UV22-2	Whole-cherry	30	24
22-2 30 WC (48 h)	*W. anomalus* UV22-2	Whole-cherry	30	48
22-2 30 WC (72 h)	*W. anomalus* UV22-2	Whole-cherry	30	72
22-2 45 WC (24 h)	*W. anomalus* UV22-2	Whole-cherry	45	24
22-2 45 WC (48 h)	*W. anomalus* UV22-2	Whole-cherry	45	48
22-2 45 WC (72 h)	*W. anomalus* UV22-2	Whole-cherry	45	72
C FW (24 h)	Without inoculation	Fully washed	-	24
C FW (48 h)	Without inoculation	Fully washed	-	48
C FW (72 h)	Without inoculation	Fully washed	-	72
1-3 15 FW (24 h)	*W. anomalus* YWP1-3	Fully washed	15	24
1-3 15 FW (48 h)	*W. anomalus* YWP1-3	Fully washed	15	48
1-3 15 FW (72 h)	*W. anomalus* YWP1-3	Fully washed	15	72
1-3 30 FW (24 h)	*W. anomalus* YWP1-3	Fully washed	30	24
1-3 30 FW (48 h)	*W. anomalus* YWP1-3	Fully washed	30	48
1-3 30 FW (72 h)	*W. anomalus* YWP1-3	Fully washed	30	72
1-3 45 FW (24 h)	*W. anomalus* YWP1-3	Fully washed	45	24
1-3 45 FW (48 h)	*W. anomalus* YWP1-3	Fully washed	45	48
1-3 45 FW (72 h)	*W. anomalus* YWP1-3	Fully washed	45	72
22-2 15 FW (24 h)	*W. anomalus* UV22-2	Fully washed	15	24
22-2 15 FW (48 h)	*W. anomalus* UV22-2	Fully washed	15	48
22-2 15 FW (72 h)	*W. anomalus* UV22-2	Fully washed	15	72
22-2 30 FW (24 h)	*W. anomalus* UV22-2	Fully washed	30	24
22-2 30 FW (48 h)	*W. anomalus* UV22-2	Fully washed	30	48
22-2 30 FW (72 h)	*W. anomalus* UV22-2	Fully washed	30	72
22-2 45 FW (24 h)	*W. anomalus* UV22-2	Fully washed	45	24
22-2 45 FW (48 h)	*W. anomalus* UV22-2	Fully washed	45	48
22-2 45 FW (72 h)	*W. anomalus* UV22-2	Fully washed	45	72

* WC = whole-cherry process, 1-3 = *W. anomalus* YWP1-3, 22-2 = *W. anomalus* UV22-2, 15 = OD15, 30 = OD30, 45 = OD45.

**Table 2:** Sensory analysis of Robusta coffee under whole-cherry fermentation.

Conditions	Cupping Notes	Score *
Control WC (24 h)	Chocolate, hazelnut, dried fruit sweet, well structure, dry mouthfeel at cool temperature	81.00 ± 0.5 ^a^
Control WC (48 h)	Bell pepper, Veggie soup, flat and mute in acid, watery	73.50 ± 0.2 ^c^
Control WC (72 h)	Cocoa, mushroom, musty, unclean aftertaste	73.75 ± 0.1 ^c^
1-3 15 WC (24 h)	Grainy, unripe fruit quality, dry finish	73.00 ± 0.8 ^c^
1-3 15 WC (48 h)	Cocoa, bitter aftertaste	73.00 ± 1.0 ^c^
1-3 15 WC (72 h)	Papery aroma, musty, rice cracker, flat at cool stage	73.25 ± 0.1 ^c^
1-3 30 WC (24 h)	Grain, glass flavor, bitter	73.25 ± 0.25 ^c^
1-3 30 WC (48 h)	Grain, cocoa, flat, lack of sweet, medium low body	72.25 ± 0.15 ^c^
1-3 30 WC (72 h)	Grain, sweet, short aftertaste	73.75 ± 0.22 ^c^
1-3 45 WC (24 h)	Popcorn, soda cracker, medium low body, short aftertaste, Low salt Low acid	76.75 ± 0.30 ^b^
1-3 45 WC (48 h)	Sweet corn, bold body, low acid, harsh finish, bittersweet	77.00 ± 0.55 ^b^
1-3 45 WC (72 h)	Chocolate, raisin, sweet, round and long aftertaste	82.00 ± 0.15 ^a^
22-2 15 WC (24 h)	Papery aroma, musty, roasted, found some acidity at hot, medium body, flat flavor at cool stage	73.50 ± 0.33 ^c^
22-2 15 WC (48 h)	Bland, mute acidity	73.25 ± 0.26 ^c^
22-2 15 WC (72 h)	Bland, bitter, medium body but harsh mouthfeel	72.75 ± 0.37 ^c^
22-2 30 WC (24 h)	Caramel, corn-liked flavor, commercial taste of robusta	75.00 ± 0.44 ^b^
22-2 30 WC (48 h)	Roasted, long bitter in aftertaste	73.50 ± 0.63 ^c^
22-2 30 WC (72 h)	Hay, grain, short aftertaste	74.00 ± 0.22 ^c^
22-2 45 WC (24 h)	Flat, cloudy, raw corn, pea garden	74.50 ± 0.35 ^c^
22-2 45 WC (48 h)	Clean, Sweet corn, hint of caramel, collapse structure when cool down.	77.00 ± 0.11 ^b^
22-2 45 WC (72 h)	Tofu, chocolate, bland, medium high body	77.50 ± 0.10 ^b^

* Data represent mean ± standard deviation (n = 5). Different letters indicate statistically significant differences among fermentation conditions (*p* < 0.05). WC = whole-cherry fermentation, 1-3 = *W. anomalus* YWP1-3, 22-2 = *W. anomalus* UV22-2, 15 = OD15, 30 = OD30, 45 = OD45.

**Table 3:** Sensory analysis of Robusta coffee under fully washed fermentation.

Conditions	Cupping Notes	Score *
Control FW (24 h)	Mute and bitter, rough mouthfeel	73.25 ± 0.15 ^c^
Control FW (48 h)	Hint of fruity, slightly bright when hot, flat at cool stage	75.50 ± 0.81 ^b^
Control FW (72 h)	Hint of fruit in aroma, grain, sharp acid	75.25 ± 0.24 ^b^
1-3 15 FW (24 h)	Brown sugar, grass, cocoa, neutral, salty show up after mid temp, baggy found in cold stage	73.75 ± 0.25 ^c^
1-3 15 FW (48 h)	Grain, peanut skin aroma, flat, mild flavor, bitterness finish (since hot stage), watery mouthfeel	72.25 ± 0.31 ^c^
1-3 15 FW (72 h)	Grain, corn, Phenolic defect, harsh, blackish, bold, powdery mouthfeel	64.5 ± 0.14 ^f^
1-3 30 FW (24 h)	Hay, corn, cornflake, saline salt	73.50 ± 0.15 ^c^
1-3 30 FW (48 h)	Nutty, grass, Brown sugar, roasted, rubber, salty, bold	73.75 ± 0.57 ^c^
1-3 30 FW (72 h)	White pepper, grain, light vanilla, hint of caramel, light sweet, smooth	75.50 ± 0.68 ^b^
1-3 45 FW (24 h)	Corn, nutty, Found strong bitter from warm to cold, astringent	71.50 ± 0.66 ^d^
1-3 45 FW (48 h)	Hay, rice, fermented soy bean, muddy, tea like body, unclean finished	69.00 ± 0.45 ^e^
1-3 45 FW (72 h)	Rubber, burn, rough, astringent, high bitter from hot to cold	70.75 ± 0.12 ^e^
22-2 15 FW (24 h)	Papers, nutty skin, leather finish, smooth, consistent in taste from hot to cold	75.50 ± 0.32 ^b^
22-2 15 FW (48 h)	Brown sugar, grain, roasted, nutty, mineral water liked in mouthfeel	74.25 ± 0.52 ^c^
22-2 15 FW (72 h)	Oily, Lubricant, roasted, cocoa powder, pungent, harsh	71.75 ± 0.12 ^d^
22-2 30 FW (24 h)	grainy, rubber, Brown Sugar, pea, salty, low acid, rough mouthfeel	72.00 ± 0.32 ^c^
22-2 30 FW (48 h)	Grassy, peanut skin, salty and rough, long saltiness and bitter in aftertaste	71.75 ± 0.33 ^d^
22-2 30 FW (72 h)	Earthy, toasted, charcoal, harsh and sandy mouthfeel, high bitter from hot to cold stage	69.75 ± 0.62 ^e^
22-2 45 FW (24 h)	Chocolate, banana, corn, savory, medium body	76.25 ± 0.84 ^b^
22-2 45 FW (48 h)	Dried fruit, cocoa, papery, bland, a bit sandy mouthfeel.	75.50 ± 0.63 ^b^
22-2 45 FW (72 h)	Nutty skin, cocoa, bitter up front, medium salt, low acid, skim milk like texture	74.25 ± 0.67 ^c^

* Data represent mean ± standard deviation (n = 5). Different letters indicate statistically significant differences among fermentation conditions (*p* < 0.05). WC = whole-cherry fermentation, 1-3 = *W. anomalus* YWP1-3, 22-2 = *W. anomalus* UV22-2, 15 = OD15, 30 = OD30, 45 = OD45.

## Data Availability

The original contributions presented in this study are included in the article/Supplementary Materials. Further inquiries can be directed to the corresponding author.

## Supplementary Materials

The following supporting information can be downloaded at: https://www.mdpi.com/article/10.3390/foods15203721/s1, Figure S1: Robusta coffee fermentation process (Whole-cherry fermentation and Fullywashed fermentation).
