# Supplementary material for: Biosafety and Fermentation Performance of Wickerhamomyces anomalus Starter Cultures in Fully Washed and Modified Whole-Cherry Fermentation of Coffea canephora
Source: Foods. 2026 Oct 19;15(20):3721. doi: 10.3390/foods15203721 (PMC13704435; doi:10.3390/foods15203721)
Supplement: Supplementary file 1 [file foods-15-03721-s001.zip › foods-4541945-supplementary.pdf]

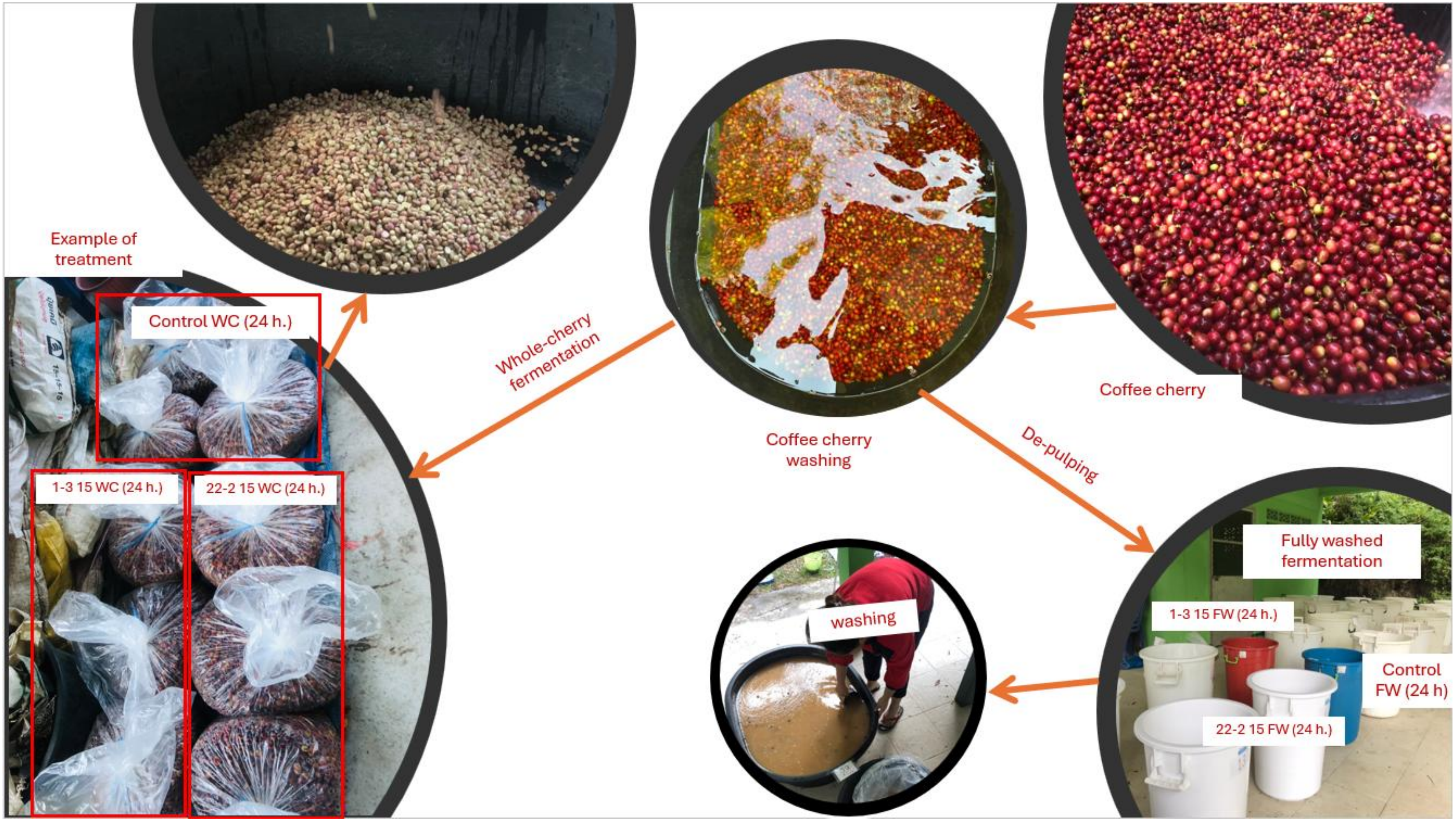

Figure S1. Robusta coffee fermentation process (Whole-cherry fermentation and Fullywashed fermentation).
